# Boron Carbide–Stainless Steel Eutectic Interactions During Severe Accidents in Sodium-Cooled Fast Reactors: From Reaction Mechanisms to Melt Relocation and Safety Implications

**DOI:** 10.3390/ma19194222

**Published:** 2026-10-05

**Authors:** Ruicong Xu, Yihua Xu, Wen Zhou, Hanyu Wang, Ravinder Kumar, Zeeshan Ahmed, Yosuke Nishimura, Xiaoxing Liu, Songbai Cheng, Kai Wang

**Affiliations:** 1 Nuclear Professional School, The University of Tokyo, 2-22 Shirane-Shirakata, Tokai-Mura, Naka-gun, Ibaraki 319-1188, Japan; 2 School of Intelligent Vehicles, Guangzhou Polytechnic University, No. 1342 Shiliang Road, Guangzhou 511483, China; 3 Department of Nuclear Engineering and Management, The University of Tokyo, 7-3-1 Hongo, Bunkyo-ku, Tokyo 113-8656, Japan; 4 MQue Co. Ltd., 2-3-21 Koraku, Bunkyo-ku, Tokyo 112-0004, Japan; 5 Sino-French Institute of Nuclear Engineering & Technology, Sun Yat-sen University, Tang-Jia-Wan, Zhuhai 519082, China; 6 College of Nuclear Science and Technology, Harbin Engineering University, Harbin 150001, China; chengsongbai@hrbeu.edu.cn

**Keywords:** eutectic melting, boron carbide–stainless steel, CALPHAD, melt relocation, sodium-cooled fast reactor, severe accident modeling

## Abstract

**Highlights:**

- Synthesizes recent and foundational evidence on B_4_C–stainless steel eutectic interactions in SFRs.
- Shows Arrhenius-type trends across methods, with method-, contact- and morphology-dependent rates.
- Separates transferable B_4_C–steel evidence from geometry-dependent and oxidation-specific findings.
- Links reaction kinetics and melt composition to boron redistribution, reactivity and IVR.
- Pinpoints gaps: sodium, irradiation and bundle-scale validation, and multicomponent melt data.
- Compiles kinetic, phase, property, experimental and model tables for SFR eutectic analyses.

**Abstract:**

Eutectic reactions between core materials strongly influence the progression of severe accidents in sodium-cooled fast reactors (SFRs). Contacting constituents, namely metallic fuel with steel cladding and boron carbide (B_4_C) absorber with stainless steel (SS), form liquid phases several hundred kelvin below the melting points of the individual materials. The resulting low-temperature liquefaction leads to early failure of cladding and control-rod structures, relocation of fuel and absorber, and changes in core reactivity, and therefore affects both the recriticality evaluation and the in-vessel retention (IVR) strategy. This focused narrative review mainly covers studies published in the last decade and earlier foundational work. It concentrates on B_4_C–SS reaction mechanisms, kinetics, thermodynamics, properties, experiments and models, with metallic fuel–cladding interaction as context. A transferability analysis distinguishes reactor-independent material-couple evidence from geometry-dependent and oxidation-specific results obtained mainly in post-Fukushima boiling water reactor studies. The principal limitations are the predominance of unirradiated small-scale tests in controlled atmospheres, incomplete multicomponent thermodynamic and transport-property data, and limited validation from single-rod to bundle and reactor scales. These uncertainties propagate through melt composition and boron redistribution to reactivity and IVR, defining priority needs for future SFR experiments and coupled modeling.

## 1. Introduction

**Figure 1:**
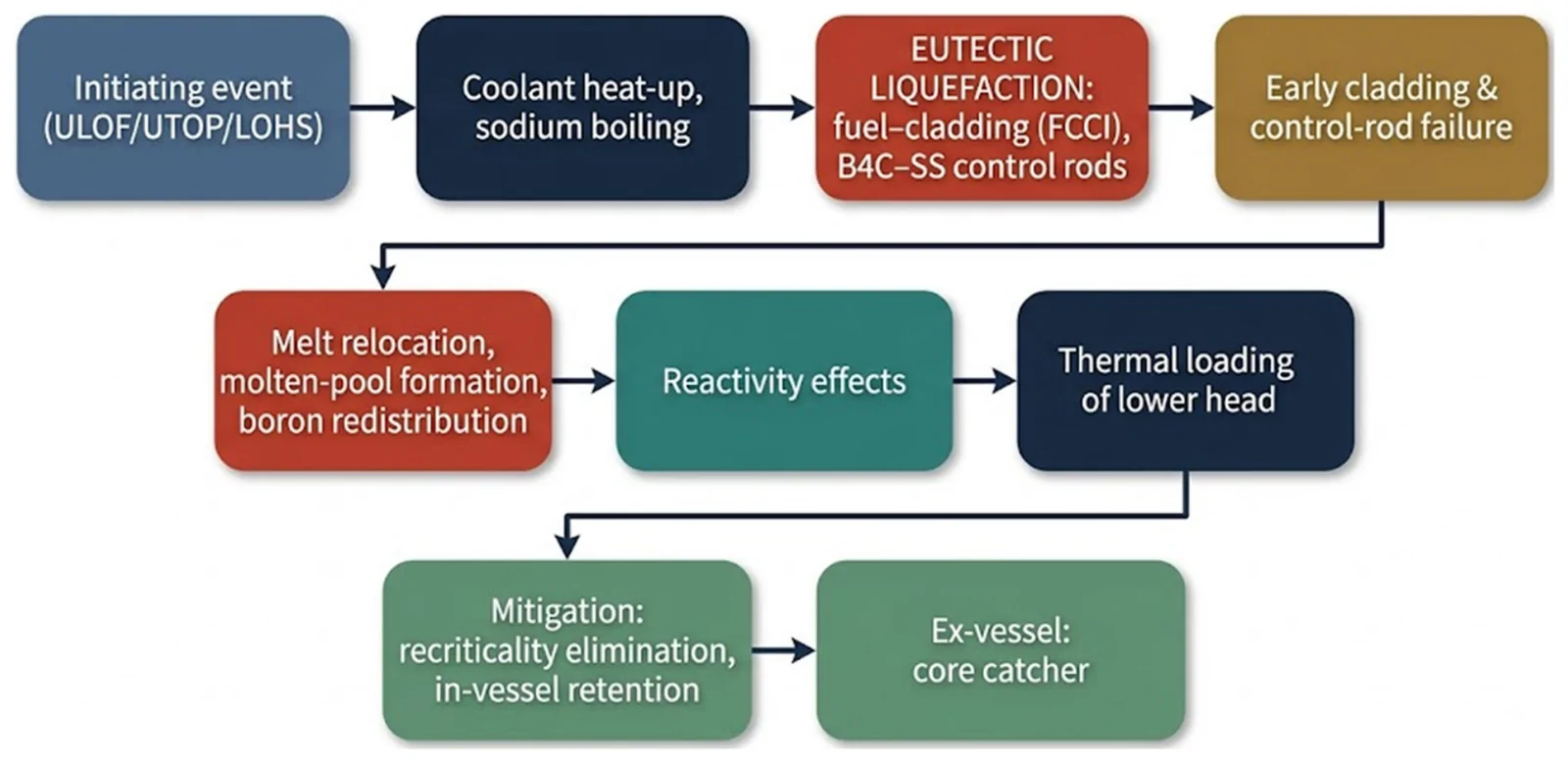
Progression of a core disruptive accident in an SFR and the position of eutectic melting within the sequence.

Sodium-cooled fast reactors (SFRs) are among the most mature Generation IV reactor concepts, with substantial operating experience from EBR-II, Phénix, BN-800, Monju, CEFR and other experimental or prototype plants [1,2]. Their appeal rests on a closed fuel cycle that can breed fissile material and transmute minor actinides, a low-pressure primary circuit, large thermal margins and favorable passive feedbacks in many designs [2,3]. Nevertheless, the safety case for any fast reactor must address low-probability, high-consequence sequences in which protection systems are assumed to fail. Unprotected loss-of-flow (ULOF), unprotected transient-overpower (UTOP) and loss-of-heat-sink transients may then lead to a core disruptive accident (CDA), in which fuel, cladding and structural materials heat beyond their stability limits, melt and relocate [4,5]. Because the fast-spectrum core is not arranged in its most reactive geometry, the central CDA safety issues are the avoidance of energetic recriticality during material motion and the retention of degraded core material inside the reactor vessel, i.e., in-vessel retention (IVR), so that the containment boundary is preserved [6,7]. Figure 1 summarizes the overall sequence.

Eutectic melting is one of the mechanisms that can change the timing and pathway of SFR accident progression. When chemically incompatible materials are in contact at elevated temperatures, interdiffusion across the interface can generate a liquid phase at a temperature far below the melting point of either constituent. Two material couples are especially important in an SFR core, but their ranges of applicability differ. The U–Fe/FCCI branch is specific to metallic-fueled SFRs, whereas B_4_C–SS absorber interaction is relevant to SFR designs that employ B_4_C control rods, including oxide-fueled concepts. The first is metallic fuel in contact with iron-based cladding. The U–Fe system contains an equilibrium low-temperature eutectic near 998 K, whereas rapid penetration of steel by molten uranium has been associated with UFe_2_ formation above approximately 1353 K (1080 °C); fuel–cladding chemical interaction (FCCI) can therefore weaken or breach the cladding before bulk steel melting [8,9]. The second couple, common to SFRs and boiling water reactors (BWRs), is the B_4_C neutron absorber and stainless steel (SS) in the control-rod cladding and surrounding structures. Although the melting point of B_4_C is about 2720 K, the B_4_C–SS couple can liquefy at approximately 1473–1523 K through boride formation and carbon redistribution [10,11]. Locally, these reactions lower the failure temperature of cladding and control-rod structures [10,12]. At the core scale, the resulting liquid can drain along remaining structures (candling, i.e., downward flow and re-freezing of melt along colder surviving structures), dissolve additional material, and redistribute both fissile fuel and neutron-absorbing boron [12,13]. Non-uniform boron redistribution changes local neutron absorption and therefore affects recriticality margins [12,14]. The composition and temperature of the relocated melt also influence the thermal and chemical loading of lower internal structures, the vessel lower head and core-catcher systems [15,16]. Integral severe-accident codes have historically represented such processes with compact melting criteria or adjustable interaction parameters [17,18].

Chemical interactions between B_4_C and stainless steel were first characterized systematically in the context of light water reactor (LWR) safety research during the 1980s and 1990s [10,19,20,21]. The Fukushima Daiichi accident of 2011, in which all affected units were BWRs with B_4_C control blades, led to renewed experimental and analytical work on absorber-rod degradation and fuel-debris chemistry [11,12,22]. A considerable part of the quantitative database and the modeling methods now applied to SFR analysis originates from this effort. In parallel, SFR-specific programs have addressed metallic-fuel FCCI [8,9], the kinetics of the B_4_C–SS reaction at CDA-relevant temperatures [23,24,25], the thermophysical properties of boron-bearing steel [14,26,27], in situ visualization of eutectic melting and relocation [13,28,29], and mechanistic simulation by particle methods [17,18,30,31]. Earlier reviews have treated metallic-fuel FCCI [8] or LWR/BWR degradation phenomenology [11,12]; the present review instead connects B_4_C–SS material-couple evidence, its transferability to SFR conditions, melt-relocation physics, and the recriticality/IVR quantities used in SFR safety assessment.

This paper therefore adopts a deliberately SFR-centered framing while treating the BWR studies as conditionally transferable evidence, and concentrates on progress over approximately the last decade, with older foundational studies retained where they define mechanisms, data correlations, or model formulations. The B_4_C–SS system is treated as the principal subject; metallic-fuel FCCI is retained as contextual evidence for metallic-fueled SFRs. Knowledge generated in the BWR context is included where it is transferable to SFR conditions, and the differences between the two applications are noted. The remainder of the paper is organized as follows: Section 2 reviews the reaction mechanisms, kinetics, thermodynamics and thermophysical properties of the relevant material systems. Section 3 describes the experimental techniques and the principal findings obtained with them, from integral bundle tests to visualization and simulant experiments. Section 4 addresses numerical simulation, from particle-based mechanistic models to integral severe accident codes and their application to IVR and core-catcher evaluation. Section 5 summarizes the outstanding issues, indicates directions for further research and presents the conclusions. This article is a focused narrative review. Relevant studies were identified through keyword searches of major bibliographic databases and academic search engines. Studies reporting reaction mechanisms, kinetic or thermophysical data, reactor-relevant degradation/relocation observations, or numerical models with a stated verification or experimental-comparison basis were retained.

## 2. Eutectic Reactions of SFR Core Materials

**Figure 2:**
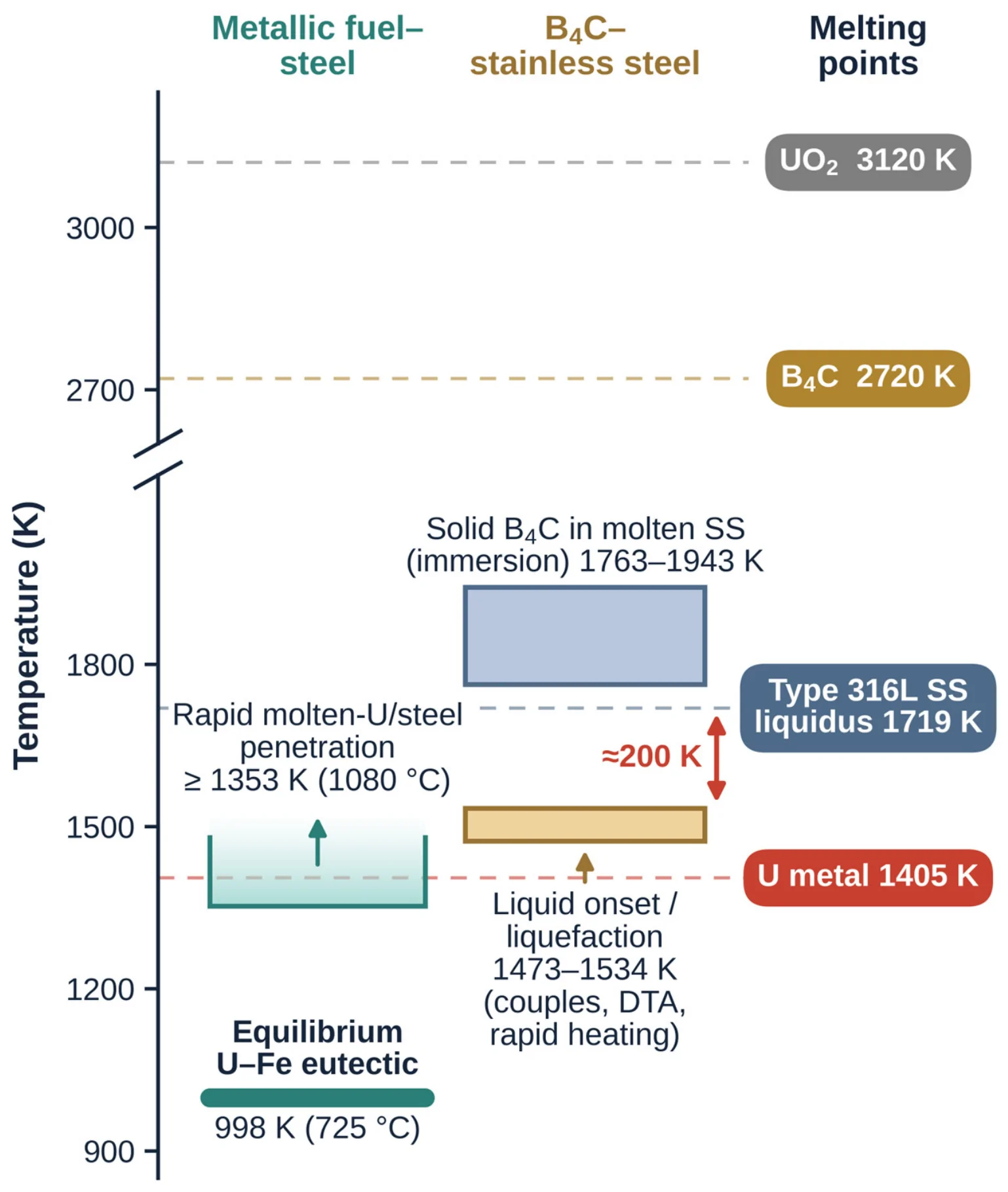
Melting points of selected core constituents compared with representative eutectic-liquefaction ranges. The U–Fe line marks the equilibrium eutectic (998 K, 725 °C) and the separate band marks the rapid molten-uranium/steel penetration regime above approximately 1353 K (1080 °C) [9]. The B_4_C–SS band denotes reported liquid-onset/liquefaction temperatures in couple, DTA and rapid-heating tests [10,19,23,32]; the solid-B_4_C/molten-SS band corresponds to the immersion tests [24]. Melting points: U [33], Type 316L SS liquidus [27], B_4_C [11] and UO_2_ [12].

Figure 2 compares the melting points of key core constituents with representative temperature ranges for eutectic liquefaction. The U–Fe line marks the equilibrium low-temperature eutectic at 998 K (725 °C), whereas rapid penetration of steel by molten uranium has been reported above approximately 1353 K (1080 °C), where UFe_2_ can form from two liquids [9]. The B_4_C–SS band (approximately 1473–1534 K) represents reported liquid-onset/liquefaction behavior in diffusion-couple, DTA and rapid-heating tests rather than a single equilibrium value and depends on test conditions [10,19,23,32]. The B_4_C–SS liquefaction onset lies approximately 200 K below the melting range of stainless steel [11] and more than 1000 K below that of B_4_C. These temperature margins define the framework within which the mechanisms, kinetics and thermodynamics of the individual systems are reviewed below. Accordingly, comparisons across studies should track material composition, steel grade, B_4_C physical form, contact state, atmosphere/coolant, heating mode and rate, and irradiation state.

### 2.1. Metallic Fuel–Cladding Chemical Interaction

FCCI in metallic fuel has been investigated since the 1960s, and the accumulated experience has been reviewed by Keiser [8]. During irradiation, the fuel slug swells and closes the fabrication gap; after contact, uranium, plutonium, zirconium and lanthanide fission products can interdiffuse with the iron, chromium and nickel of the cladding. Lanthanides accumulate near the fuel periphery and form brittle interaction layers, while uranium and iron form low-melting U–Fe mixtures including UFe_2_ and U_6_Fe [8,9]. Under normal operation the interaction is treated as gradual cladding wastage; during accident heating the same chemistry can provide a low-temperature path to cladding failure. Quantitative FCCI behavior additionally depends on fuel composition, cladding alloy, irradiation history and lanthanide redistribution [8]. For example, out-of-pile couples showed liquid formation with Fe- or Ni-based cladding alloys at temperatures as low as ~823 K (550 °C) for U–Pu–Fs fuel, whereas U–Pu–Zr fuel was credited with a fuel–cladding eutectic temperature of ~1088 K (815 °C), and the nitrogen content of the cladding influenced the interdiffusion behavior [8]. The binary U–Fe description alone is therefore insufficient for prediction of irradiated-fuel behavior.

**Figure 3:**
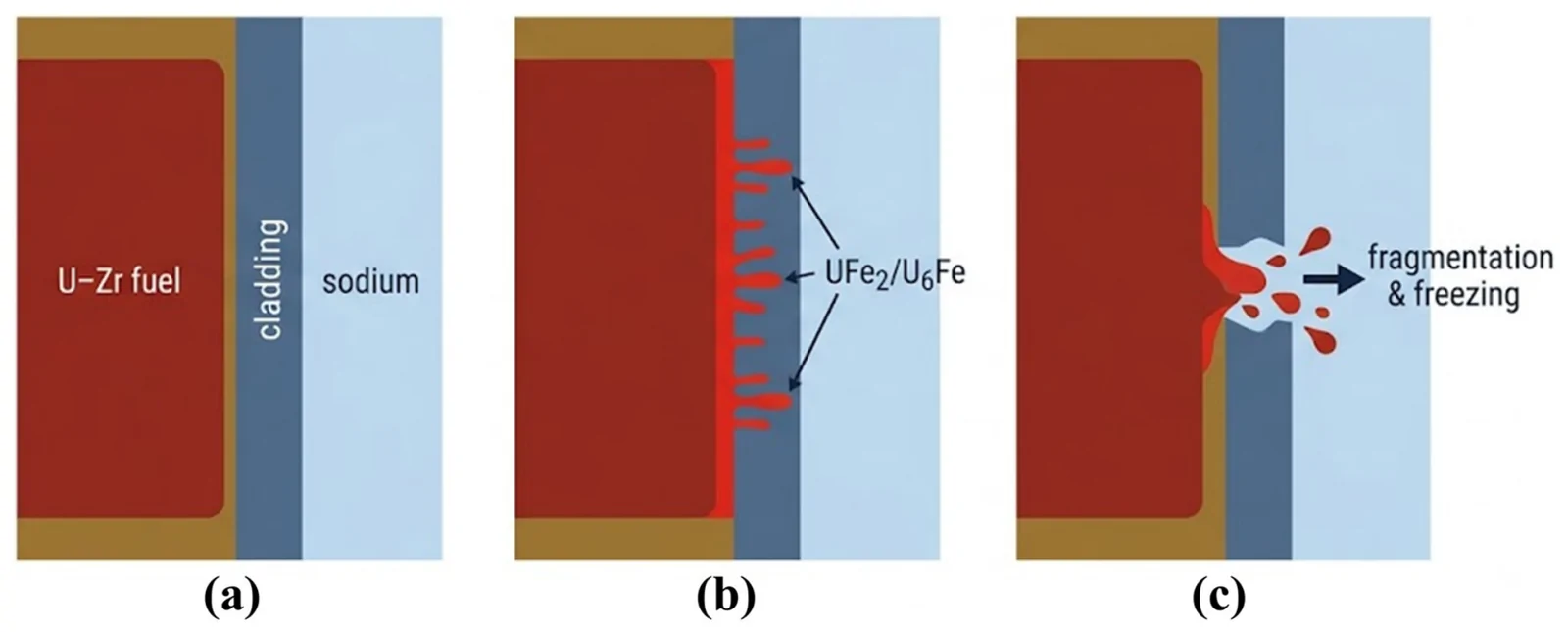
Schematic of eutectic penetration of metallic fuel into steel cladding: (**a**) fuel–cladding contact after swelling; (**b**) interdiffusion and eutectic penetration with formation of UFe_2_ and U_6_Fe; and (**c**) cladding breach and melt release into the sodium channel.

The behavior beyond cladding breach was examined by Kim et al. [9], who released molten uranium into an HT-9M cladding tube and characterized the products by scanning electron microscopy and energy-dispersive spectroscopy (SEM/EDS). Eutectic penetration initiated at the fuel–cladding boundary and progressed into the cladding wall until failure, as illustrated schematically in Figure 3. In the failed region the original cladding geometry was no longer recognizable, and UFe_2_ and U_6_Fe were identified as the principal phases. The released fuel–steel mixture entered the sodium channel, where its fragmentation and freezing determine the subsequent reactivity and coolability. These observations form part of the experimental basis used to formulate metallic-fuel models in the SAS4A code, which tracks the evolving composition and relocation of fuel and cladding components during transients [5]. The Kim et al. experiment represents a single-pin molten-uranium/HT-9M geometry; transfer to accident-scale behavior therefore requires separate treatment of transient heating, sodium cooling, multi-pin geometry and material relocation.

With regard to accident tolerance, comparative assessments indicate that the high thermal conductivity and low stored energy of metallic fuel result in cladding failure below the sodium boiling point and in coolable configurations for a wide range of transients, despite the lower melting temperature relative to oxide fuel [1]. Analyses comparing oxide, nitride and metallic fuels under ULOF and UTOP conditions show that metallic fuel can begin to melt earlier during overpower transients because of the lower melting point of uranium (1405 K), and that the resulting uranium melt can breach the cladding through eutectic interaction before the cladding itself melts [33]. The quantitative treatment of these trade-offs requires the eutectic kinetics discussed below.

### 2.2. Reaction Mechanism and Layer Structure of the B_4_C–SS System

The framework for the B_4_C–SS reaction was established by compatibility studies in the late 1980s and 1990s [10,19,20,34]. Hofmann et al. [10] examined B_4_C against Type 316 SS and Zircaloy; for the B_4_C/SS couple, reaction-zone growth followed a parabolic law below extensive liquefaction, and the growth rate was about two orders of magnitude higher than that of B_4_C/Zircaloy above 1273 K. The B_4_C/SS specimens liquefied rapidly above approximately 1523 K, whereas the B_4_C/Zircaloy couple liquefied only at substantially higher temperatures. Nagase et al. [19] annealed B_4_C/Type 304 SS couples isothermally between 1073 and 1623 K, resolved the multi-layer reaction zone, and identified a discontinuity in the temperature dependence of the parabolic rate constant between 1473 and 1498 K, corresponding to liquid-phase formation at the interface.

**Figure 4:**
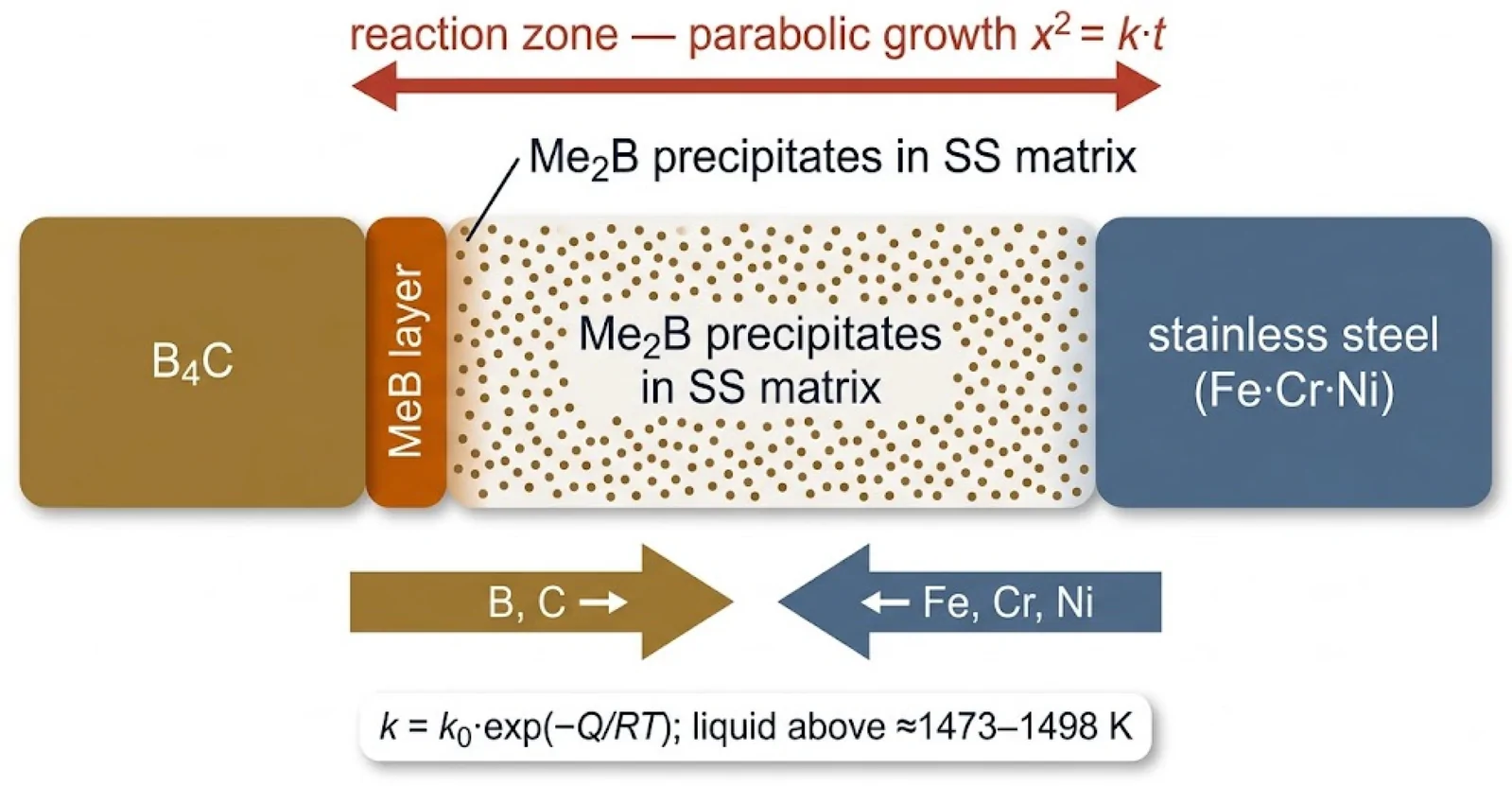
Reaction-zone structure of the B_4_C–stainless steel diffusion couple and the associated element fluxes.

The mechanistic interpretation is that of a diffusion couple. Boron and carbon diffuse from the B_4_C into the steel, while iron, chromium and nickel diffuse in the opposite direction, and boride layers nucleate and grow in the sequence dictated by local thermodynamics, as illustrated in Figure 4. Veshchunov and Hofmann [20] measured concentration profiles in couples annealed at 1273–1473 K by Auger electron spectroscopy and X-ray microanalysis, described the main reaction layer as Me_2_B precipitates (Me = Fe, Cr, Ni) in a steel matrix, and derived the chromium diffusion coefficients in the constituent phases, providing a quantitative description of layer growth. Related modeling of multicomponent interactions was reported in [35]. The parabolic description assumes planar, diffusion-controlled growth with stable layers; it ceases to apply when the liquid phase becomes convective, when the geometry departs from a flat couple, or when additional materials participate in the reaction.

Steam oxidation is not part of the intrinsic B_4_C–SS interaction under the baseline in-vessel SFR sodium environment. Oxidation-specific LWR/BWR evidence is therefore treated separately in Section 2.6 and in the transferability discussion of Section 3.6.

### 2.3. Reaction Kinetics

**Figure 5:**
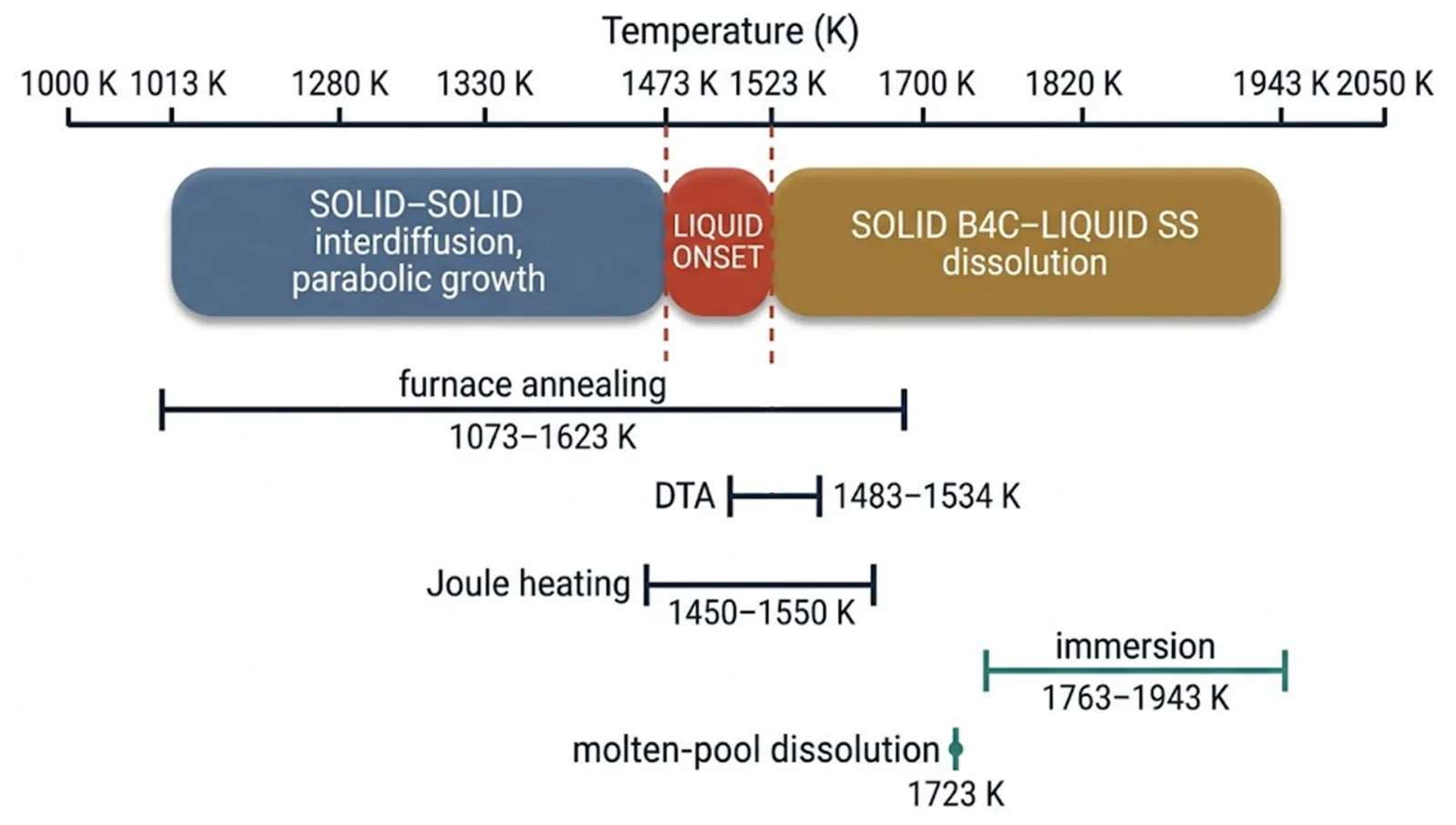
Temperature regimes of the B_4_C–stainless steel reaction and the ranges covered by the principal kinetic measurements.

Recent work has broadened the kinetic database across several reaction regimes, which are summarized in Figure 5 together with the temperature ranges of the principal measurement campaigns. Three regimes are distinguished: solid B_4_C against solid steel below the liquefaction onset, solid B_4_C against liquid steel above it, and the dissolution of B_4_C into a bulk steel melt.

**Figure 6:**
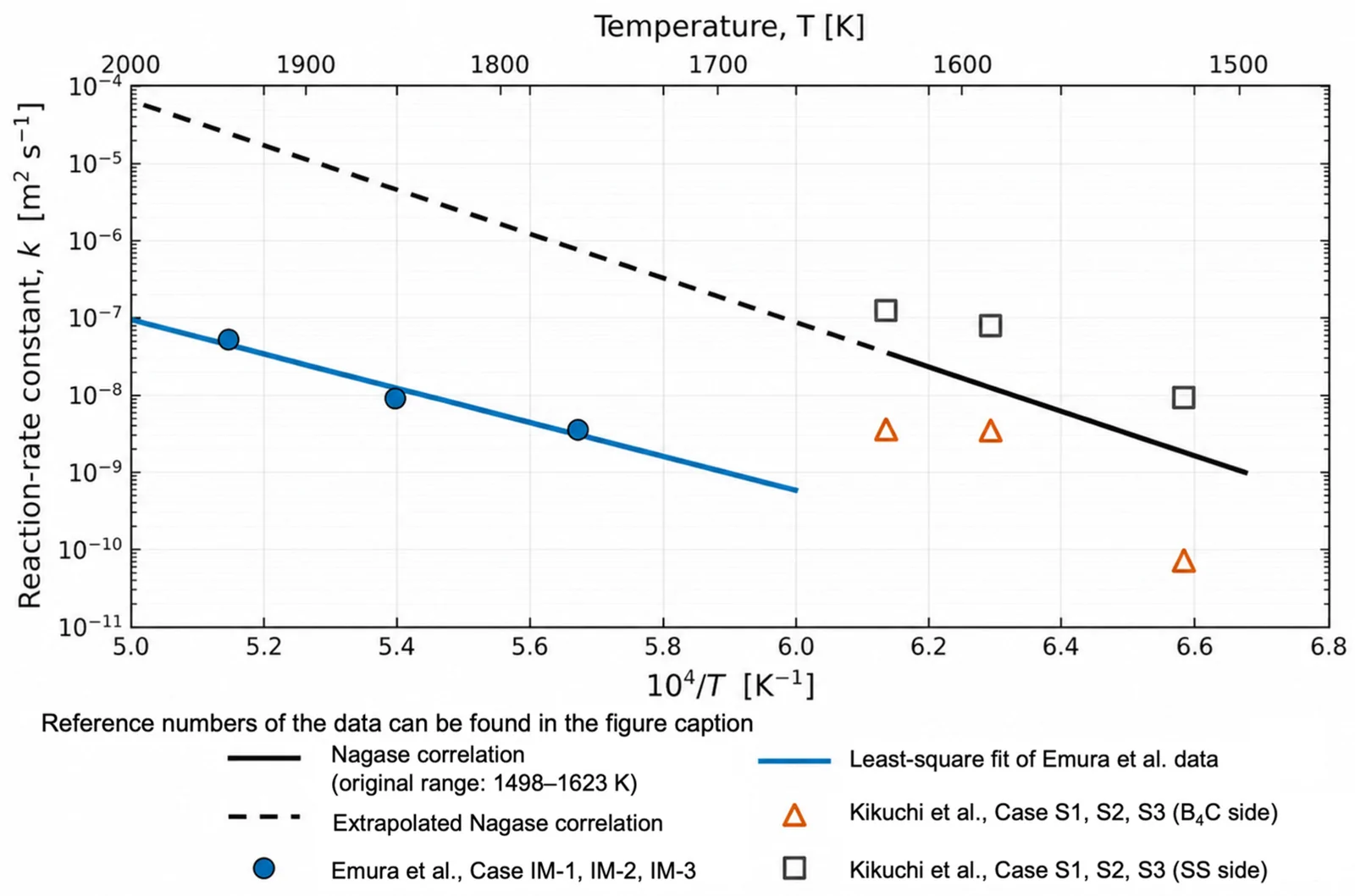
Comparison of reaction rate constants for the B_4_C–SS system (drawn on the basis of the immersion data [24], the solid–solid reaction-test data of Kikuchi et al. re-evaluated on the B_4_C and SS sides in [23], and the correlation of Nagase et al. [19]). The plotted quantities should therefore be interpreted as a comparison of temperature trends rather than as one universal rate constant; the measured side of the interface, geometry and transport regime differ among the datasets.

For the solid–solid regime, Kikuchi et al. [23] performed differential thermal analysis (DTA) of B_4_C and Type 316L SS pellet samples up to 1773 K at heating rates of 2.5–10 K min^−1^. Relevant data are shown in Figure 6. Endothermic peaks associated with the eutectic reaction appeared between 1483 and 1534 K and shifted systematically to higher temperatures with increasing heating rates; the apparent activation energy (511 ± 119 kJ mol^−1^) and pre-exponential factor (ln(A/s^−1^) = 34.6 ± 9.7) obtained by the Kissinger method were comparable to values derived from earlier thinning experiments, and electron-probe microanalysis of the interdiffusion layer provided validation data for the initial stage of the reaction. Bai et al. [32] investigated the effect of rapid heating on the B_4_C–Type 316L SS system and reported that the effective eutectic temperature increased from 1476 K at a heating rate of 80 K min^−1^ to 1503 K at 110 K min^−1^, while the diffusion-layer thickness decreased from 1.64 to 1.13 mm; the phases Fe_2_B, Cr_2_B and CrB identified by SEM/EDS, X-ray diffraction (XRD) and electron backscatter diffraction (EBSD) were consistent with CALPHAD calculations.

For the solid–liquid regime, as shown in Figure 6, Emura et al. [24] immersed B_4_C pellets in molten stainless steel at 1763–1943 K and evaluated rate constants from the reduction in pellet thickness. The temperature dependence was similar to that of the lower temperature solid–solid reaction, but the absolute rate constants were smaller than values extrapolated from solid–solid correlations by two or more orders of magnitude, reflecting differences in the evaluated thickness, material side and transport regime. Kanno et al. [36] investigated the reaction of molten Type 316L SS with B_4_C at 1723 K under Ar and Ar–H_2_O atmospheres and found that the dissolution rate was not affected by water vapor, that Fe and Cr borides precipitated at the interface, and that interfacial carbon precipitation reduced the B_4_C dissolution rate.

A quantitative method based on Joule heating was developed by Hong et al. [25], in which the steel specimen is heated by an electric current and the reaction-layer growth is measured optically with high spatial resolution. Rate constants were obtained in the range 1450–1550 K, where data had been scarce, and were consistent with literature values. The method was subsequently applied to compare B_4_C powder and B_4_C pellets in contact with SS tubes [37]. The reaction with powder was considerably faster than with pellets, and the pellet case exhibited a two-layer reaction zone consisting of a thin uniform MeB layer adjacent to the B_4_C and a thicker layer of MeB precipitates in the steel. Since control rods employ both material forms, this geometry effect is directly relevant to accident analysis. Complementary soaking experiments by Matsuura et al. [38] quantified the attack of Zircaloy by molten SS–B_4_C, extending the database to mixed-material contacts in BWR geometries.

Table 1 shows that furnace annealing, DTA, immersion, Joule heating and molten-pool dissolution measurements now cover most of the temperature range relevant to early B_4_C–SS interaction. The datasets agree in showing Arrhenius-type temperature trends, but they should not be collapsed into a single universal rate constant. Absolute values depend on the B_4_C form, contact geometry, which side of the interface is used to define the reaction thickness, and whether the melt remains diffusion-controlled or becomes convective. Two systematic effects still require quantitative closure: the difference between powder and pellet forms [37], and the influence of interfacial carbon accumulation on B_4_C dissolution [36]. Nearly all determinations were performed on unirradiated materials under controlled atmospheres. Quantitatively, reported apparent activation energies range from about 250–380 kJ mol^−1^ for solid-state layer growth below ≈1473 K [10,19] to 453–549 kJ mol^−1^ above the liquid-phase onset [19]; the first-order DTA onset analysis gives 511 ± 119 kJ mol^−1^ [23], and granular contacts give 623 ± 279 kJ mol^−1^ with large contact-related scatter [39]. Across the onset, the parabolic rate constant increases by about two orders of magnitude between 1473 and 1498 K [19], and the immersion constants lie two or more orders of magnitude below extrapolated solid–solid values [24]. In addition, rapid heating shifts the effective onset from 1476 K at 80 K min^−1^ to 1503 K at 110 K min^−1^ [32], while granular B_4_C/Type 304L SS contacts have shown parabolic rate constants approximately one order of magnitude lower than tighter pellet/powder contacts [39]. Heating rate, absorber morphology and contact state should therefore be treated as explicit applicability variables of kinetic correlations rather than as secondary descriptors.

### 2.4. Phase Relations and Thermodynamic Assessment

The identification of reaction products and the prediction of melting behavior require phase-equilibrium data for the Fe–B–C system and its extensions with chromium and nickel. Sudo et al. [40] measured phase relations in the boron- and carbon-rich region of the Fe–B–C ternary system, for which data had been insufficient. Shibata et al. [39] examined couples of granular B_4_C and Type 304L SS, the material combination used in Japanese BWR control rods, and resolved the reaction region into two to four layers with pronounced composition variations. Takano et al. [41] prepared solidified melts of B_4_C, stainless steel, Zr, ZrO_2_ and (U,Zr)O_2_ by arc melting and determined the phase assemblages relevant to fuel-debris characterization. Phase-diagram information for the B–Cr binary system is available in [42]. The thermodynamic calculations in the cited studies used the TCFE12 steel/Fe-alloy database for the B_4_C–Type 316L SS couple [32] and Thermo-Calc with the TAF-ID nuclear-fuel database for the UO_2_–B_4_C–steel and oxidized SS–B_4_C systems [43,44]. The former does not cover U-bearing phases, whereas the reliability of the latter for the UO_2_–B_4_C–steel system was itself being tested against the 2173 K experiments [43]. Extrapolation is therefore the least reliable in B- and C-rich multicomponent Fe–Cr–Ni–B–C–U compositions, where direct measurements remain sparse.

**Figure 7:**
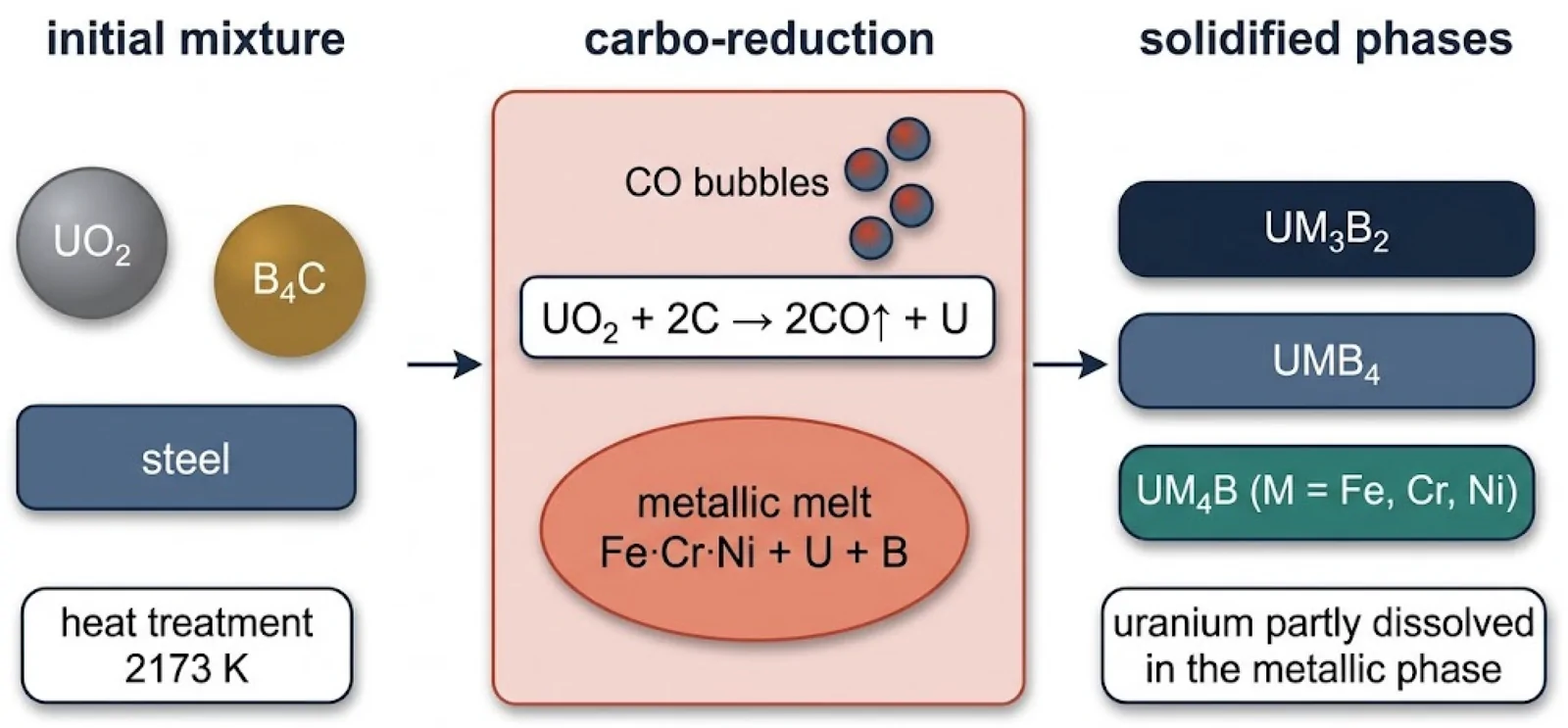
Schematic summary of the UO_2_–B_4_C–steel interaction at 2173 K, showing carbo-reduction, uranium dissolution into the metallic phase and the formation of mixed borides (drawn on the basis of the results reported in [43]).

The boride and carbo-boride phases reported across these studies, (Cr,Fe)_2_B, CrB, Fe_2_B and (Cr,Fe)_23_(C,B)_6_, have been identified by an expanding set of techniques, as shown in Table 2. Fukai et al. [45] applied Raman spectroscopy with multivariate curve resolution together with SEM/EDS to distinguish the products of the B_4_C–SS reaction from those of the FeB–SS reaction, and reported an interfacial layer corresponding to amorphous carbon or iron borides in the B_4_C–SS case, whereas no distinct layer formed between FeB and SS. The role of carbon in the multi-material system was examined by Garrigue et al. [43], who heat-treated mixtures of UO_2_, B_4_C and steel at 2173 K and identified a carbo-reduction reaction, UO_2_ + 2C → 2CO + U, by which uranium from the fuel dissolves into the metallic melt and forms mixed borides of the type UM_3_B_2_, UMB_4_ and UM_4_B (M = Fe, Cr, Ni), as illustrated schematically in Figure 7. These results indicate that binary descriptions of the absorber and fuel systems are not sufficient for the late accident phase, in which several materials interact simultaneously. Table 2 distinguishes phases identified directly by microscopy, diffraction or spectroscopy from those supported by thermodynamic interpretation, and also lists the reaction-layer phases reported for diffusion-couple, Joule-heating and radiative-heating tests.

The solidified-melt and steam-oxidation data summarized in Table 2 provide additional experimental anchor points for the thermodynamic discussion [44,46]. Thermodynamic calculations based on the CALPHAD method underpin the interpretation of these experiments [47]. Assessed databases for the U–Zr–O and related corium systems [48], together with experimental phase-equilibrium studies of corium–steel interactions [49,50,51,52], provide the wider framework within which the B_4_C-bearing subsystems are being integrated. The multicomponent Fe–Cr–Ni–B–C–U description remains incomplete in the boron- and carbon-rich composition ranges, which is one motivation for the targeted measurements cited above. Equilibrium calculations do not provide reaction rates, and must be combined with the kinetic data of Section 2.3 for transient analysis. CALPHAD results are therefore used here to interpret phase stability and liquid formation, not as direct experimental phase identification.

**Table 2:** Experimentally identified and calculation-supported phases in B_4_C–stainless steel(–fuel) systems and their accident relevance.

System/Condition	Directly Identified Phases	Experimental Methods	Calculation Role	Evidence Basis	Accident-Relevant Consequence	Ref.
B_4_C–Type 316 SS diffusion couples; 1273–1473 K	Me_2_B (Me = Fe, Cr, Ni) precipitates in SS matrix	AES, XMA	Diffusion-controlled growth model (no CALPHAD)	Measured concentration profiles + kinetic model	Defines solid-state reaction-layer growth before liquefaction	[20]
Granular B_4_C–Type 304L SS; Ar; 1453–1523 K	Two to four reaction layers; (Fe,Cr) borides with strong composition gradients	SEM, EDS, WDS	None required for primary identification	Measured phases/compositions	Loose granular contact slows reaction (BWR control-rod geometry)	[39]
B_4_C pellet–Type 304 SS tube; Joule heating; Ar; 1450–1550 K	Thin uniform MeB layer adjacent to B_4_C; MeB precipitates in steel	SEM/EDS	None required for primary identification	Measured phases	Explains slower pellet than powder reaction	[37]
B_4_C–Type 316L SS; rapid heating 80–110 K min^−1^ (onset 1476–1503 K)	(Cr,Fe)_2_B, CrB, Fe_2_B	SEM/EDS, XRD, EBSD	TCFE12 (CALPHAD) consistency check	Measured phases + calculation support	Constrains liquid onset and boride formation under transient heating	[32]
B_4_C–SS and FeB–SS couples; Type 316L/304 SS; heated to ~1503–1518 K	Fe_2_B/FeB-related products; amorphous-carbon/boride interfacial layer	Raman (MCR), SEM-EDS	None required for primary identification	Measured spectroscopy/microanalysis	Shows carbon/boride interfacial chemistry relevant to rate limitation	[45]
Solidified SS–B_4_C melts; Type 304/316L SS, 1–5 mass% B_4_C	γ-Fe, (Cr,Fe)_2_B; (Cr,Fe)_23_(C,B)_6_ above ~3 mass% B_4_C	PXRD, SEM-EDS, TG-DTA	Phase-diagram interpretation of solidification/re-melting path	Measured phases	Links B_4_C inventory to solidus/re-melting and retained boron phases	[46]
B_4_C powder/pellet in SS cladding; Ar; radiative heating 1423–1645 K	B_0.9_Cr_0.9_Fe_1.1_, (Cr,Fe)_2_B, γ-Fe, (Cr,Fe)_23_(B,C)_6_, (Cr,Fe)_5_B_3_	XRD, XPS, SEM-EDS	None required for primary identification	Measured phases	Boron retained in the relocated eutectic melt	[53,54]
UO_2_–B_4_C–steel; 2173 K	UM_3_B_2_, UMB_4_, UM_4_B (M = Fe,Cr,Ni) and U in metallic phase	EDS, EBSD, EPMA	TAF-ID (v11) equilibrium calculation supports carbo-reduction and phase interpretation	Measured phases + thermodynamic interpretation	Demonstrates multicomponent coupling and redistribution of U/B in late accident melt	[43]
Type 304 SS–B_4_C; steam, 1373 K	Fe–B oxide, Fe–Cr–O, Fe–O layers	Metallography	TAF-ID phase-stability (oxygen-potential) calculation	Measured layers + calculation support	BWR oxidation-specific boron retention; not a baseline in-vessel SFR chemistry	[44]

### 2.5. Solidified Products and Corrosion–Erosion

The characterization of solidified SS–B_4_C mixtures is required both for fuel-debris retrieval at Fukushima Daiichi and for the late phase of SFR accident sequences. Sumita and Kobayashi [55] investigated the corrosion–erosion of solid stainless steel by SS–B_4_C melt at 1573 K and observed infiltration of the melt into the grain boundaries of the solid steel; the infiltration length increased parabolically with immersion time, the rate depended on the grain size, and the grain-boundary diffusion coefficient of boron was estimated to be of the order of 10^−5^ cm^2^ s^−1^. Sumita et al. [46] characterized solidified SS–B_4_C mixtures over the B_4_C content range expected in Fukushima Daiichi debris by powder XRD, SEM-EDS and thermogravimetry–differential thermal analysis, and reported that γ-Fe and (Cr,Fe)_2_B are the major phases below about 3 mass% B_4_C, with (Cr,Fe)_23_(C,B)_6_ appearing at higher contents; the solidification path and the re-melting behavior were described on this basis. These non-oxidizing solidified-product and corrosion–erosion observations can inform SFR relocation chemistry when composition is matched, but they do not reproduce sodium heat transfer or reactor-scale geometry.

### 2.6. Steam Oxidation as BWR-Specific Evidence

Itoh et al. [44] exposed solidified Type 304 SS–B_4_C alloy to steam at 1373 K for 60–240 min and observed a three-layer oxide structure consisting of an internal oxidation zone containing B and Cr oxides, an intermediate Fe–Cr–O/Fe–B–O layer and an external Fe–O layer. The results indicate that boron can be retained in absorber-derived debris as Fe–B oxide and that internal oxidation limits its release from the solid phase. Together with the corium-chemistry studies discussed in Section 2.4, these investigations support Fukushima Daiichi debris characterization. These steam-dependent oxidation products are not direct inputs to baseline in-vessel SFR calculations; they are retained as BWR-specific evidence for oxidizing or ex-vessel conditions.

### 2.7. Thermophysical Properties of B_4_C-Bearing Steel

Melt-relocation modeling requires property data for steel containing dissolved boron and carbon. Takai et al. [26] synthesized homogeneous SS–5 mass% B_4_C samples by hot pressing and measured solid-phase properties: the density was lower than that of Type 316L SS and decreased with temperature, the specific heat was slightly higher, and the thermal conductivity was lower than that of Type 316L SS up to 1273 K.

**Figure 8:**
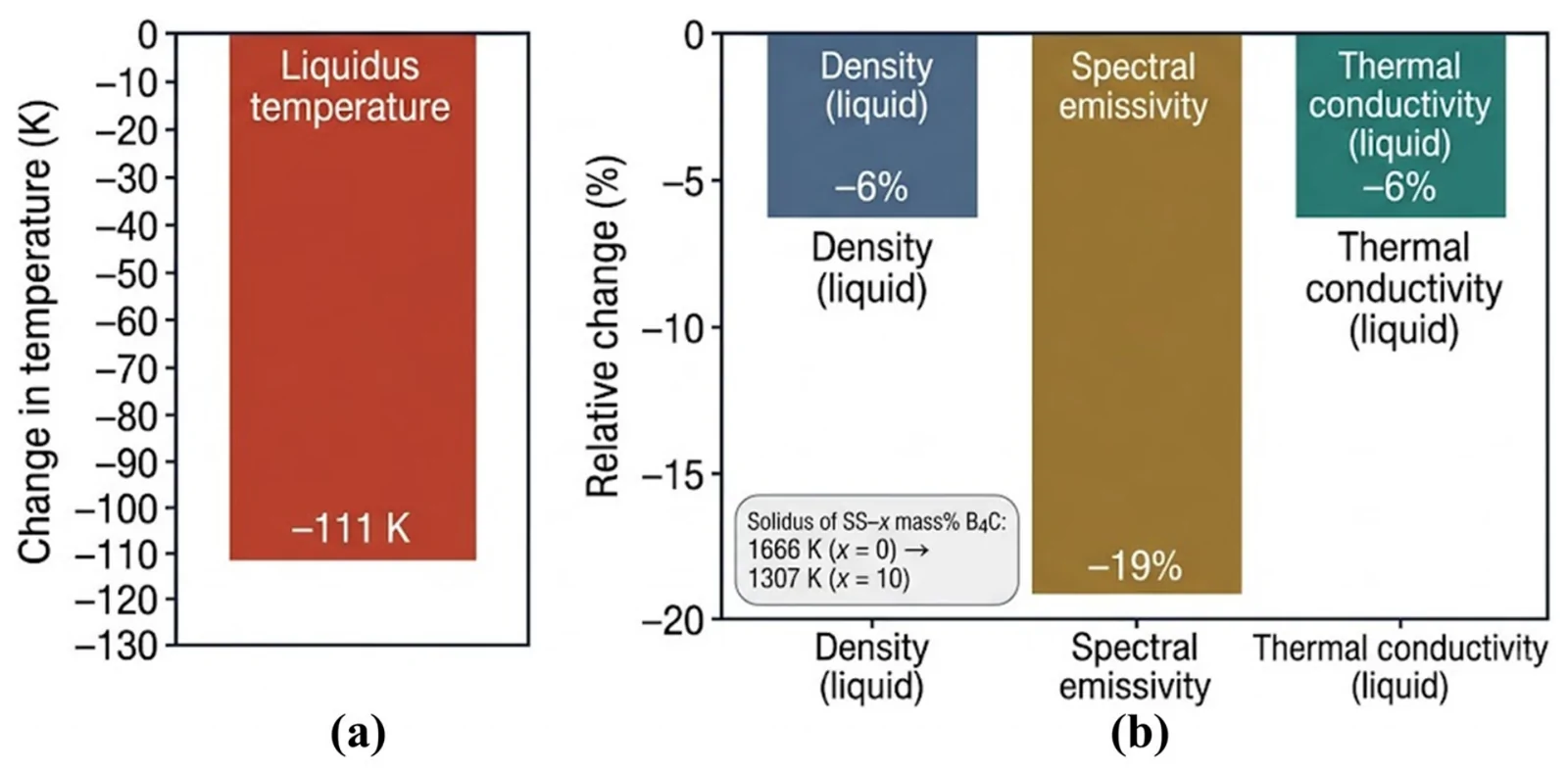
Measured changes in the properties of molten Type 316L SS upon addition of 5 mass% B_4_C; the inset note gives the solidus depression over 0–10 mass% B_4_C (drawn on the basis of the results reported in [14,27]): (**a**) phase-transition temperature; (**b**) dimensionless property changes.

For the liquid phase, Fukuyama et al. [27] used electromagnetic levitation in a static magnetic field to measure molten Type 316L SS and SS–5 mass% B_4_C without crucible contamination and over a range that included undercooling. Adding 5 mass% B_4_C shifted the liquidus downward by 111 K; at the liquidus it also lowered the density, normal spectral emissivity and thermal conductivity by approximately 6%, 19% and 6%, respectively. The later composition-series work [14] showed that increasing B_4_C from 0 to 10 mass% progressively depresses the solidus from 1666 to 1307 K and reduces the density. In contrast, the liquidus passes through a minimum around 2.5 mass% B_4_C, and the measured surface tension is controlled more strongly by dissolved sulfur than by the nominal B_4_C addition. These results are summarized in Figure 8. Reference correlations for stainless steel properties [56] and levitation measurements on related melts [57] support the evaluation of these data. Viscosity data for B_4_C-bearing melts are available from Nishi et al. [58], who measured molten Type 316L SS containing 0–7.0 mass% B_4_C by the oscillating-crucible method over composition-dependent ranges within 1613–1793 K (measurement error below ±8%) and found that viscosity increases with B_4_C content. The normal spectral emissivity, specific heat capacity and thermal conductivity have also been measured for 0–10 mass% B_4_C [59]. Remaining gaps concern higher B_4_C fractions, higher temperatures and multicomponent U/Zr-bearing melts, and the sulfur sensitivity of surface tension should be treated as an explicit input uncertainty. The property data recommended for relocation models are compiled in Table 3.

## 3. Experimental Investigations of Eutectic Melting and Relocation

### 3.1. Integral and Bundle-Scale Tests

Integral experiments established the influence of eutectic reactions on bundle degradation before separate-effect programs quantified the underlying kinetics. In the SFR context, the SCARABEE-N in-pile program examined the melting and propagation behavior of subassemblies under total instantaneous blockage, including the liquefaction of cladding and fuel and the attack of neighboring structures [60]. For BWR geometries, the DF-4 experiment in the ACRR reactor [61], the XR2-1 metallic-melt relocation experiment [62] and the CORA bundle tests with B_4_C absorber [63] showed that control-blade materials fail and liquefy at approximately 1523 K, considerably below the temperatures at which the UO_2_–Zircaloy interaction becomes significant [64,65], and that the resulting melt relocates downward and interacts with channel-box and cladding materials. The Phébus FP tests demonstrated that fuel-bundle degradation proceeds at temperatures below the fuel melting point through liquid-phase formation [66,67,68]. Syntheses of this body of work with reference to the Fukushima Daiichi accident are given by Steinbrück [11] and Kurata et al. [12], and the OECD/NEA BSAF benchmark compared integral-code predictions of the accident progression in the three damaged units [22]. Lower-head penetration failure driven by eutectic melting between a melt pool and the control-rod drive housing was investigated experimentally by Yamashita et al. [69] in the ELSA-1 test, which identified eutectic melting as a possible failure mechanism of the BWR lower-head boundary. In the discussion below, SCARABEE-N is treated as direct SFR evidence, whereas DF-4, XR2-1, CORA, Phébus, BSAF and ELSA-1 are used as LWR/BWR mechanistic analogues for onset, relocation mode and melt–structure interaction rather than as directly transferable SFR thresholds.

### 3.2. Dynamic Visualization Experiments

Time-resolved observation of the B_4_C–SS reaction was introduced by Ueda et al. [28], who placed B_4_C powder between stainless steel plates and recorded the reaction region optically during heating. The temperature, the reaction area and the maximum reaction-layer thickness were obtained as functions of time, in contrast to post-test examination of quenched specimens. In a subsequent study [29], a Type 304 tube filled with B_4_C powder was heated above the eutectic temperature (1473 K) to simulate a control rod, and the initial relocation of the eutectic melt was classified into three modes: film formation, droplet formation without collapse, and droplet formation with collapse, with the mode depending on the cladding thickness. This classification links the interfacial reaction to the subsequent melt transport and has been used in the interpretation of later experiments and simulations. Quantitative thresholds inferred from these images remain experiment-specific because temperature measurement/thermocouple placement, optical spatial calibration and edge identification introduce uncertainty, and systematic inter-facility reproducibility data are still limited.

### 3.3. Joule-Heating Experiments

The Joule-heating method of Hong et al. [25,37], described in Section 2.3 with respect to its kinetic results, also constitutes a visualization technique: the specimen is heated rapidly and locally by the current, and the growth of the reaction layer is followed optically with high resolution. The method requires an electrically continuous specimen and concentrates on heat generation where the electrical resistance is largest, which restricts the accessible geometries. Within these restrictions it provides rate data with short test times, and the derived rate constants were checked against furnace-based literature values [25]. Unlike accident-relevant external or radiative heating, Joule heating generates heat within the electrically conducting steel and can localize temperature gradients where electrical resistance is greatest; therefore, transfer of the measured rates requires comparable local temperature fields and contact states.

### 3.4. Radiative-Heating Experiments

A concentrated radiative-heating facility was developed at the University of Tokyo in cooperation with JAEA to observe melting and relocation in rod-scale B_4_C–SS control-rod specimens [13]. Radiative heating imposes a thermal boundary condition comparable to that of a degrading core, in which the control rod is heated from outside by its surroundings, and permits optical access during long-duration melting. The current tests are nevertheless single-rod experiments with unirradiated materials in argon; sodium heat transfer/wetting, irradiation effects and bundle interactions are not represented and are treated here as central limitations.

**Figure 9:**
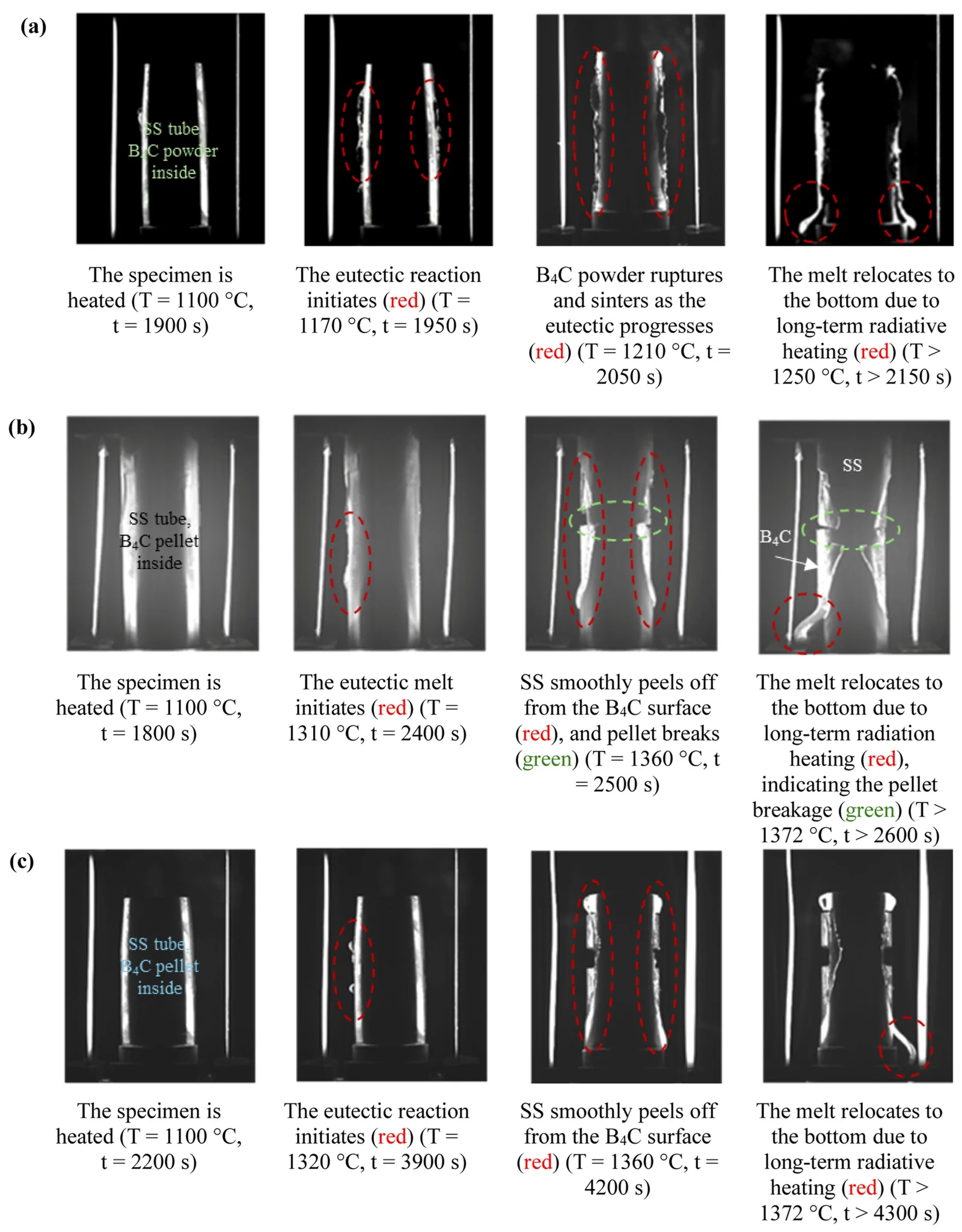
Melt progression sequences of B_4_C–SS control-rod specimens under radiative heating: (**a**) powder-type specimen; (**b**,**c**) pellet-type specimens (reproduced from [54]). Specimens: Type 304 SS tube (outer diameter 21.4 mm, wall thickness 0.6 mm, length 45 mm) filled with B_4_C powder (particle size approximately 30–35 µm) or containing a cylindrical B_4_C pellet (diameter 20 mm, length 45 mm).

Experiments with B_4_C pellets in SS claddings between 1423 and 1645 K identified two failure processes: separation of the SS cladding from the B_4_C pellet, producing a melt droplet that detaches later, and fracture of the B_4_C pellet into several pieces, interpreted as thermal-stress cracking [13,53]. The pellet configuration exhibited a higher eutectic onset temperature than the powder configuration and, within the tested conditions, showed little sensitivity to the heating rate [53]. With B_4_C powder, sintering was observed and the cladding failed by rupture rather than by smooth peeling or separation [53,54]. The melt progression sequences observed for powder- and pellet-type specimens are shown in Figure 9. The observed cladding separation, droplet formation and pellet breakup are direct experimental observations; attribution of pellet breakup specifically to thermal-stress cracking is an interpretation of the thermal history rather than an independently measured fracture-stress result.

XRD, X-ray photoelectron spectroscopy (XPS) and SEM-EDS analyses of the solidified melts identified the boride phases B_0.9_Cr_0.9_Fe_1.1_, (Cr,Fe)_2_B, γ-Fe, (Cr,Fe)_23_(B,C)_6_ and (Cr,Fe)_5_B_3_ [53,54]. A subsequent study combined uniform radiative heating with a one-dimensional configuration to isolate the diffusion of boron in the eutectic melt, and evaluated the transport parameters against a numerical model [70]. The candling behavior observed in these tests, in which melt formation, drainage and re-freezing proceed over extended periods, provides relocation data under thermal conditions that furnace and Joule-heating experiments do not reproduce. The measured boron concentration evolution in the later uniform-heating study is experimental, whereas the extracted transport coefficient is model-dependent. The morphology trends are qualitatively consistent with independent furnace studies of powder, pellet and granular B_4_C [19,39] and with Joule-heating measurements from the same research group [37], but reproducibility across independent radiative-heating facilities has not yet been established.

### 3.5. Simulant-Material Experiments

Experiments with low-melting-point simulant couples complement the prototypic-material programs by permitting parametric studies under directly observable conditions. Mustari et al. [17] reported Pb–Sn contact-melting experiments conducted at CRIEPI, which served as the validation basis for early particle-method simulations. Xiong et al. [71] from Sun Yat-sen University developed an apparatus consisting of a sample holder and a visible resistance furnace and investigated the Pb–Sn couple (eutectic temperature 456 K) at 463–483 K under contact pressures of 0.43–1.15 MPa and several contact geometries. The reaction temperature and the contact pressure increased the reaction rate measurably, whereas the effect of contact geometry was small. A subsequent study [72] employed Sn particles in contact with Pb pellets to represent the interaction between fuel debris and reactor structures; the influence of particle diameter and geometry was traced to the competition between contact area and contact pressure, and block–block configurations reacted faster than particle–pellet configurations. The interaction between a molten pool and the vessel lower head was simulated with liquid bismuth and solid lead rods (eutectic temperature 398.5 K), and the effects of pool temperature, rod temperature, gas pressure, load and baffle configuration on the reaction-rate constant were quantified [73]. The contact-melting mechanism underlying these experiments has also been studied in layered film systems [74].

Simulant couples do not reproduce the boride chemistry of the prototypic system, and the transfer of the measured rate correlations to reactor materials requires scaling arguments that have not yet been established. Their principal contribution is the provision of parametric datasets with well-defined boundary conditions for the validation of the transport components of numerical models. The complementarity of the experimental approaches is shown in Table 4. Meaningful transfer of simulant rate correlations would require similarity in the relevant heat/phase-change timescales, diffusion-versus-convection mass transfer, wetting/contact angle and capillary balance, and contact pressure/real contact area. Simulants can therefore validate transport and contact-melting components, but not prototypic boride chemistry, phase formation or boron redistribution.

### 3.6. Transferability of BWR-Derived Evidence to SFR Conditions

Because the B_4_C–SS material couple is common to BWR and SFR control systems, the extensive post-Fukushima BWR database provides an important starting point for SFR analysis. Transferability, however, should be judged at the level of the quantity being used rather than by reactor type alone. In this context, direct transfer means that the governing local mechanism is expected to remain unchanged once steel grade, B_4_C form, contact condition, material composition and heating history are matched; it does not mean that a numerical value obtained in a BWR experiment can be inserted unchanged into an SFR calculation. The transferability categories used in this review are summarized in Table 5: directly transferable at the local material-couple level, conditionally transferable after parameter matching, qualitative/model-validation only, and not transferable to baseline in-vessel SFR conditions.

At the local material-couple scale, results from B_4_C–Type 304/316 SS diffusion couples tested in argon or a vacuum are the most transferable. Counter-diffusion of B and C into the steel and of Fe, Cr and Ni toward the B_4_C, the sequence of boride-layer formation, parabolic solid-state layer growth and the onset of interfacial liquid formation are intrinsic features of the B_4_C–SS system [10,19,20,23,24,25,32]. Likewise, phase assemblages and composition-dependent solidus and liquidus temperatures, density, heat capacity, thermal conductivity and surface tension measured for solidified or molten SS–B_4_C alloys can be used in SFR models when composition and impurity levels are matched [14,26,27,40,41,46]. The principal qualifications are the effects of steel grade, sulfur content, B_4_C physical form and the presence of U- or Zr-bearing constituents during later accident stages. Solid–liquid dissolution data for molten Type 316L SS in contact with B_4_C are also transferable at the local scale when temperature, alloy composition, convection and interfacial carbon precipitation are represented [24,36]; the H_2_O component of such tests should not be treated as the baseline SFR environment.

By contrast, experiments that reproduce control-rod geometries are more strongly dependent on geometry and boundary conditions, regardless of whether they were developed in BWR or SFR contexts. BWR-specific plate, tube and bundle studies [28,29,39,61,62,63,64,65,66,67,68] show that contact configuration, packing characteristics and cladding geometry can influence eutectic onset, reaction progression and subsequent melt relocation. SFR-oriented separate-effect and rod-scale studies [13,37,53,54,70] likewise demonstrate that the B_4_C physical form, contact state, packing or porosity, heating mode and cladding geometry can shift the apparent eutectic onset and alter the failure and relocation modes. These experiments are therefore most valuable for identifying mechanisms and trends and for model validation, rather than for direct transfer of rate constants, failure times, melt masses or flow paths across reactor designs. In particular, quantitative values obtained from BWR-specific experiments should not be applied directly to pellet-based SFR control rods because the absorber form, rod geometry, thermal boundary conditions, surrounding materials and coolant environment differ. Conversely, even SFR-oriented single-rod experiments [13,37,53,54,70] do not yet reproduce the full effects of sodium, irradiation, bundle-scale interactions or reactor-scale accident transients, and their quantitative results should therefore be used with corresponding qualification. The morphology/contact effects quantified in Section 2.3 (e.g., rate constants about one order of magnitude lower for granular contacts [39]) preclude a single universal LWR/BWR-to-SFR correction factor.

Steam-oxidation results are the least directly transferable to in-vessel SFR analysis. In BWR and other LWR environments, oxidation of B_4_C or solidified SS–B_4_C melt can produce boron oxides and boric acids, Fe–B–O and Fe–Cr–O layers, hydrogen, and oxidation-dependent retention or release of boron [11,44,75,76,77,78]. These reactions can modify melt composition, rheology and subsequent material interactions, but they are not representative of the normal SFR in-vessel environment, where the degrading core is surrounded by sodium rather than steam. They remain relevant to ex-vessel stages, post-relocation oxidation, or explicitly justified oxidant-ingress scenarios.

Overall, reactor type is not the primary discriminator for intrinsic phase relations or composition-dependent properties; the decisive factors are material composition, physical form, scale and boundary conditions. BWR-derived evidence should therefore be used hierarchically in SFR analysis: local non-oxidizing reaction and property data may support quantitative models after parameter matching; geometry-dependent relocation and bundle-scale observations should be used mainly for mechanistic interpretation and model validation; and steam-oxidation data should be applied only to scenarios in which an oxidizing environment is physically credible. A further unresolved gap is the sodium environment itself: sodium can alter heat transfer, wetting, fragmentation/freezing and relocation, and may change interfacial conditions even when the intrinsic B_4_C–SS chemistry is unchanged. The present database is too sparse to quantify these effects as a single correction. Qualitatively, because the B_4_C–SS liquid-onset temperature (approximately 1473 K) lies well above the in-core sodium boiling point (about 1200 K [1]), extensive absorber liquefaction is expected mainly after local dryout or voiding, consistent with B/Fe eutectic formation in the disrupted core region in the CDA scenario for the Japan sodium-cooled fast reactor (JSFR) [6]; sodium would then act mainly on the subsequent relocation, for example through quenching, fragmentation and freezing of melt entering sodium-filled regions [9]. These expectations remain to be confirmed experimentally.

## 4. Numerical Simulation of Eutectic Melting and Melt Progression

### 4.1. Moving Particle Semi-Implicit Method

**Figure 10:**
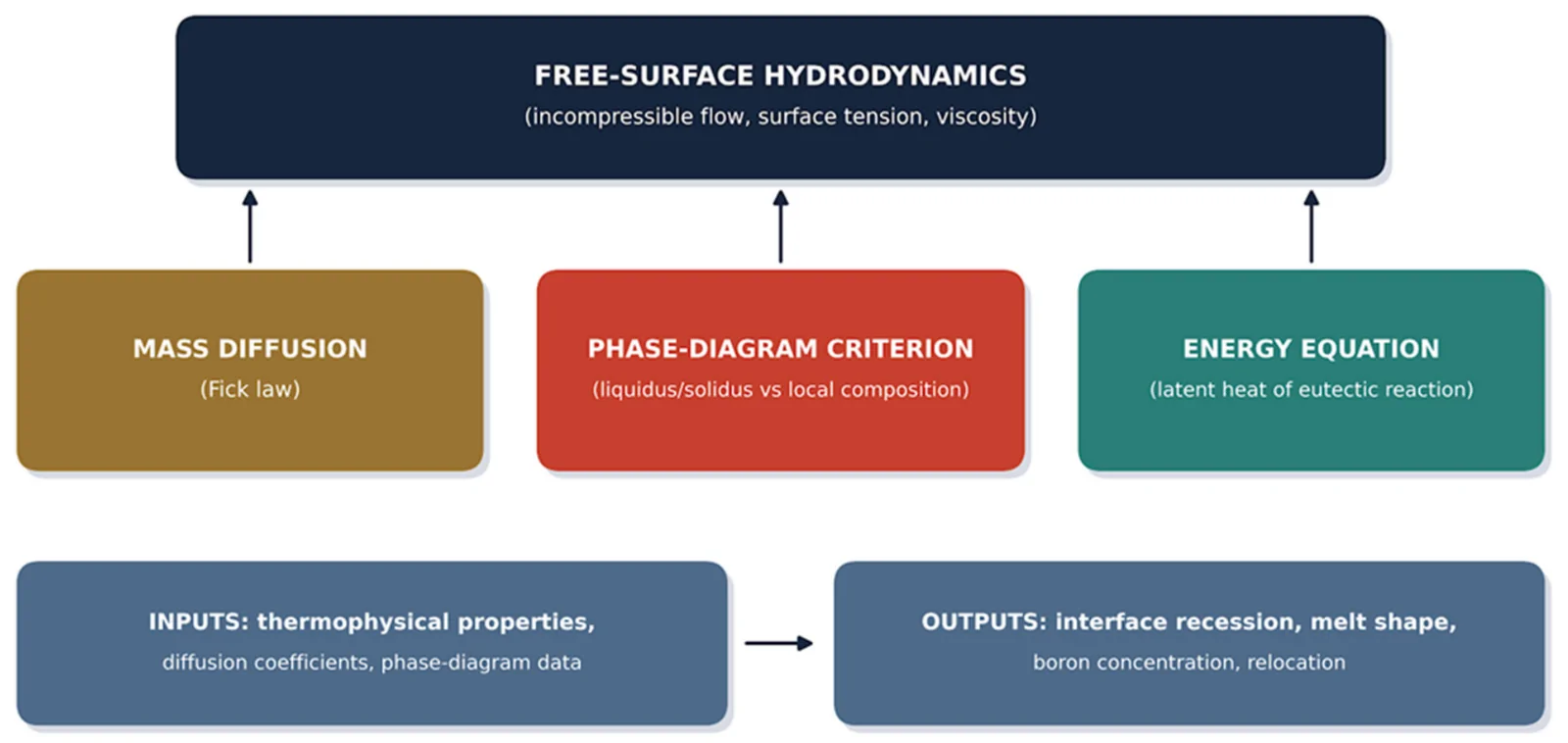
Structure of particle-method models (MPS/SPH) for eutectic melting, with their input data and validation basis.

Mesh-based methods can become cumbersome when melting and relocation produce large interface deformation, breakup and free-surface flow. For this reason, particle methods have been applied to mechanistic eutectic-melting simulations. The general structure of these models, shown in Figure 10, consists of a free-surface hydrodynamics solver coupled with a mass-diffusion equation, a phase-diagram-based melting criterion and an energy equation with latent-heat treatment.

Mustari et al. [17] applied an improved moving particle semi-implicit (MPS) code to three-dimensional Pb–Sn experiments, with mass diffusion described by Fick’s second law and the melting criterion taken from the binary phase diagram. The calculated penetration rates were compared with measurements at 478 and 498 K, and the simulation indicated that the Sn component liquefies before the Pb component. Related MPS developments for melting and stratification problems are reported in [79,80]. Zhu et al. [18] extended the approach with a heat-transfer formulation based on the phase diagram to account for the heat release and absorption of the eutectic reaction. The model was verified against one-dimensional melting solutions; the simulated U–Fe reaction in two semi-infinite domains followed the parabolic law, and the calculated penetration rates were consistent with results from the Transient Reactor Test Facility (TREAT) experiments. Ahmed et al. [30] revised the surface-tension and viscosity treatments of the standard MPS method and introduced a eutectic-reaction criterion based on the Fe–B phase diagram; the resulting model simulated boron diffusion and melt relocation in the B_4_C–SS system in two and three dimensions and was compared with the radiative-heating experiments of Section 3.4.

In many current particle-method studies, the diffusion coefficient remains an effective parameter adjusted to reproduce measured reaction rates, and the results depend on the inter-particle distance, which sets the resolved length scale. These models therefore reproduce the experiments used for calibration and interpolate between them. Predictive application will require independently measured transport coefficients and explicit resolution-convergence tests. Agreement with analytical or one-dimensional melting solutions is treated as verification, whereas comparison with independent experiments is treated as validation. When a diffusion coefficient is fitted to the same experiment later used for comparison, the exercise is calibration rather than independent validation. Predictive use also requires particle/mesh convergence, parameter-identifiability checks and sensitivity analysis because a fitted coefficient can compensate for unresolved convection, interfacial resistance or contact geometry.

### 4.2. Smoothed Particle Hydrodynamics

Zhang et al. [31] simulated the dissolution of solid B_4_C in liquid stainless steel in three dimensions using smoothed particle hydrodynamics (SPH). Their formulation combined an implicit incompressible pressure solver with viscosity, surface-tension, heat-transfer and Fickian-diffusion models. The JAEA isothermal immersion experiments at three temperatures [24] were reproduced when the diffusion coefficients were fitted to an exponential temperature dependence. The simulations indicated that melt convection can accelerate the eutectic reaction relative to purely diffusive transport, and the particle-size effect was examined analytically. This convective contribution is absent from quiescent-diffusion models and may explain part of the scatter among reported rate constants. Because the directly relevant SPH literature is presently limited, the fitted exponential temperature dependence of the diffusion coefficient is treated here as calibration to the JAEA immersion data rather than as a direct physical prediction. Table 6 compares the SPH and MPS formulations.

### 4.3. Integral Severe Accident Codes

Whole-plant analysis relies on integral codes in which local interactions are represented by compact models. Albright et al. [81,82] benchmarked the two material-interaction models available in MELCOR V2.2, the eutectics model and the interactive-materials model, for a BWR-3 Mark-I station blackout under boundary conditions representative of Fukushima Daiichi. The eutectics model produced faster accident progression, larger debris masses in the lower plenum and higher debris temperatures at lower-head failure, whereas the interactive-materials model delayed degradation and allowed greater oxidation and hydrogen generation. The different outcomes obtained from the same boundary conditions show how strongly integral predictions depend on the chosen material-interaction model. Transient ASTEC simulations of the core-melt composition at Fukushima Daiichi are reported in [83]. For metallic-fuel SFRs, the SAS4A code includes fuel and cladding relocation models and eutectic-interaction models developed for PGSFR analyses [5]. The EUCLID/V2 code with the HEFEST-FR module simulates melt retention and cooling in the core catcher of sodium-cooled reactors by solving the two-dimensional heat-conduction problem with melting for the catcher tray materials [16]. Suzuki et al. [6] provide the corresponding JSFR CDA scenario study, in which phenomenological diagrams were used to organize the possible IVR-failure pathways and the design measures intended to prevent them. For SFR interpretation, the important distinction is that SAS4A directly represents metallic fuel/cladding degradation and relocation, whereas EUCLID/V2–HEFEST-FR addresses sodium-reactor core-catcher melting/heat conduction; neither should be treated as the same material-interaction model as MELCOR/ASTEC. Table 7 summarizes the roles and available validation/sensitivity evidence.

The integral codes represent eutectic phenomena by simplified melting criteria for reasons of computational cost. The benchmark results of [81,82] indicate that the parameters of these compact models should be derived from the mechanistic data reviewed in Section 2 and Section 3, with quantified uncertainties, rather than treated as adjustable inputs. A practical uncertainty-transfer route is to infer probability distributions for kinetic/property parameters from separate-effect datasets, retain correlations associated with common composition/contact variables, and propagate these distributions through the compact integral-code model by sensitivity or sampling calculations, as in the MELCOR uncertainty analysis of [82].

### 4.4. In-Vessel Retention and Core-Catcher Analysis

The evaluation of melt stabilization measures depends on the composition, temperature and timing of melt arrival, which are outputs of the eutectic-affected relocation phase. Czarny et al. [15] performed two-dimensional axisymmetric computational fluid dynamics simulations of a corium jet impinging on a sacrificial core-catcher material and reproduced the pool effect observed in experiments, in which the accumulated melt pool limits the ablation of the sacrificial layer; sensitivity calculations addressed the influence of jet diameter, temperature and velocity. Material effects on in-vessel retention, including the influence of melt composition on the heat-flux distribution, were analyzed for LWR configurations in [84,85], and comparative reviews of melt progression in SFRs and pressurized water reactors are given in [7]. In these analyses, the boron content of the melt, determined by the extent of the B_4_C–SS reaction during relocation, affects the solidus and liquidus temperatures, density, stratification behavior and the distribution of fuel-bearing material in the melt pool. This connects the mechanistic work of Section 2 to retention evaluation. For SFR IVR/core-catcher assessment, the LWR corium studies cited above are therefore used only as general melt-transport/heat-flux analogies unless sodium and boron-bearing composition are explicitly matched. The safety pathway emphasized here, consistent with the boron-discharge and B/Fe-eutectic factors identified in the JSFR CDA scenario [6], is: B_4_C reaction extent → boron-bearing melt fraction/composition → solidus/liquidus and density/viscosity/interfacial properties → relocation/stratification and spatial boron inventory → recriticality and IVR metrics. Ex-vessel fuel–coolant interaction, including steam-explosion modeling [86], lies outside the scope of this review.

## 5. Remaining Issues, Future Prospects and Conclusions

### 5.1. Remaining Issues

Three categories of main remaining issues can be identified from the previous works. The highest-priority database uncertainty for recriticality is the extent and spatial redistribution of boron-bearing eutectic melt. The available kinetic and property data were obtained predominantly on unirradiated materials, in small specimens, and they do not yet span the accident-relevant temperature envelope: B_4_C–SS liquid formation begins at approximately 1473–1534 K [10,19,23,32]; CDA evaluations summarized in [58] indicate that degraded-core temperatures near control-rod assemblies may approach about 2273 K, whereas kinetic data extend only to 1943 K [24] and liquid-property data to about 1800–1900 K [27,58]. Data for irradiated fuel and absorber materials, for prototypic control-rod geometries, and for simultaneous multi-material interactions are limited; the UO_2_–B_4_C–steel experiments at 2173 K [43] represent one of the few studies of the latter type. The reported rate constants also retain systematic differences associated with material form, as shown by the powder–pellet comparison [37], and with interfacial phenomena such as carbon accumulation [36]. Available thermophysical-property data remain incomplete in both composition space and property coverage. Several liquid-state properties have been measured over 0–10 mass% B_4_C [14,59] and viscosity over 0–7.0 mass% B_4_C [58], whereas the solid-phase data remain limited to about 5 mass% B_4_C [26]. Moreover, the melt composition evolves continuously during an accident and may include additional fuel and structural-material constituents. These limitations directly control how much boron enters the mobile melt and where it relocates.

The second priority concerns relocation timing and IVR through coupled model/property uncertainty. The eutectic composition of the melt determines the boron distribution and thus the reactivity of the disrupted core; the melt properties determine the relocation and stratification; and the latent heat determines the local thermal balance. Few models treat these aspects simultaneously. The particle methods incorporate phase-diagram criteria and latent-heat treatments [18,30], and the SPH results indicate a non-negligible influence of melt convection on the reaction rate [31], but the transport coefficients remain calibrated rather than predicted, and the coupling to neutronics has not been implemented. At the integral-code level, the MELCOR benchmark [81,82] demonstrates that the choice of the material-interaction model alone changes the predicted accident progression, including the timing of lower-head failure and hydrogen generation.

The third priority concerns scaling, validation and uncertainty transfer between model levels. The simulant-couple experiments [71,72,73] provide clean parametric data but differ chemically from the prototypic system; the visualization experiments [13,25,28] approach prototypic materials and geometries but at single-rod scale and in inert atmospheres; the particle-method simulations reproduce individual experiments with fitted coefficients [30,31]. Traceable validation chains from simulant to prototypic materials, from single-rod to bundle scale, and from mechanistic models to integral codes have not yet been established. A defensible chain requires independently measured parameters, resolution-converged mechanistic calculations, blind or withheld-condition comparisons, and uncertainty-bounded mapping from separate-effect data to integral-code outputs.

**Figure 11:**
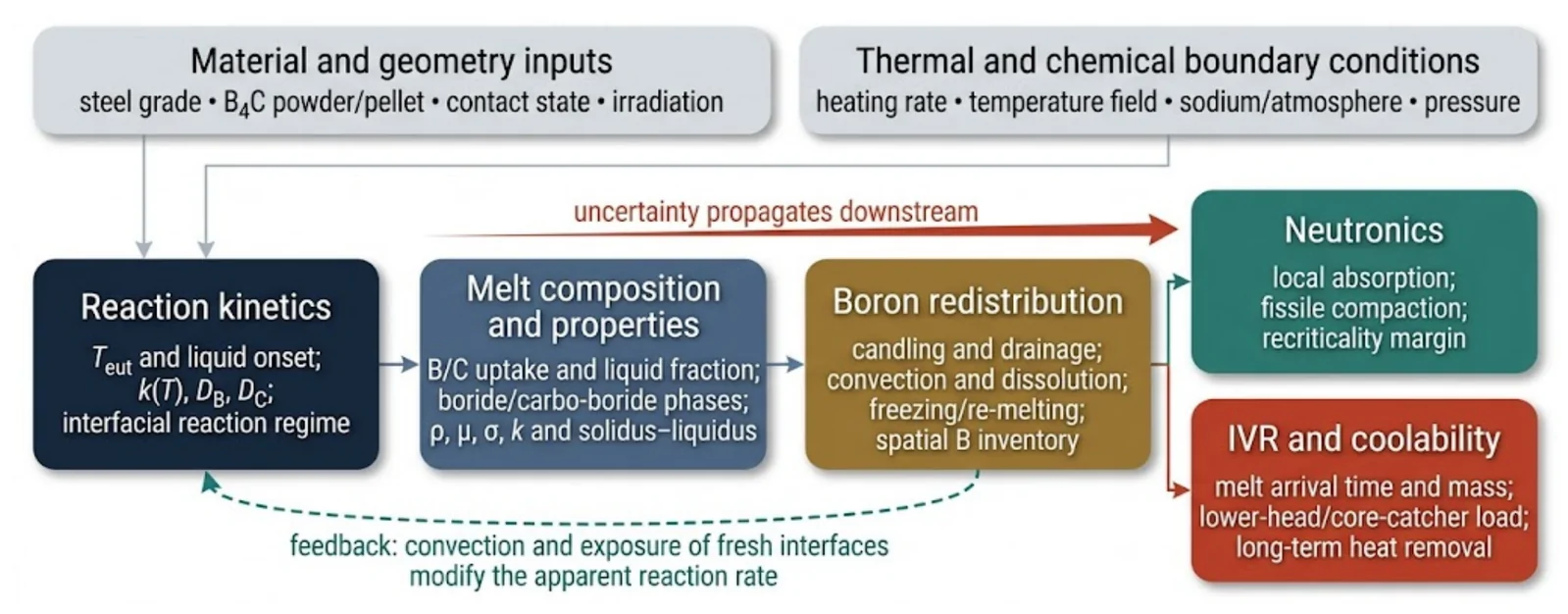
Uncertainty network from measurable B_4_C–SS reaction and property inputs through reacted mass, melt composition, boron relocation and melt-arrival conditions to SFR reactivity and in-vessel-retention outputs. Relocation/convection feeds back to the interfacial reaction, and common composition variables correlate multiple property inputs.

These three issue categories are not independent. They form a directed uncertainty chain in which an uncertainty introduced at the reaction stage is carried into the system-level safety metrics. As summarized in Figure 11, uncertain onset temperatures, rate constants and transport coefficients determine the amount and composition of eutectic liquid; those quantities set the thermophysical properties and the extent of boron entrainment; and the resulting spatial boron inventory and melt-arrival conditions enter the recriticality and IVR calculations. Convection and exposure of fresh interfaces provide feedback from relocation to the apparent reaction rate. The measurable inputs include onset-temperature distributions, kinetic/transport coefficients, B_4_C form/contact variables, thermodynamic/liquid-fraction data, density/viscosity/surface tension and coolant heat-transfer conditions. Intermediate outputs are reacted mass, liquid composition, boron entrainment, relocation velocity and melt-arrival temperature; the final safety outputs are spatial boron inventory, reactivity response and IVR/core-catcher thermal margins. Because relocation exposes fresh interfaces and changes convection/contact, feedback and correlated composition-dependent properties prevent a strictly one-way propagation.

### 5.2. Future Prospects

Near-term priorities are to harmonize kinetic definitions and uncertainty reporting across furnace, DTA, immersion, Joule- and radiative-heating methods; extend viscosity/interfacial-property measurements; and perform resolution-converged particle-method calculations with transport coefficients determined independently of the validation test. The validation target should be uncertainty-bounded reproduction of onset, reacted thickness/mass and relocation history in a withheld condition rather than a fitted reproduction of the calibration case.

Medium-term priorities are SFR-representative experiments that combine transient heating rate, pellet/granule form, prototypic rod/bundle geometry and sodium heat-transfer/wetting conditions, with irradiation effects introduced where feasible. Inter-laboratory comparisons should quantify temperature calibration, image-based spatial uncertainty and reproducibility. In parallel, CALPHAD/experimental work should constrain B- and C-rich Fe–Cr–Ni–B–C–U(–Zr–O) compositions and provide liquid-fraction/property data for multicomponent melts. A successful validation target is the blind prediction of boron redistribution and melt-arrival conditions at rod/bundle scale with stated uncertainty bounds.

Longer-term work should couple validated thermochemistry/kinetics and particle/CFD relocation models to integral severe-accident and neutronic calculations. Compact-code parameters should be derived from the mechanistic database as probability distributions with retained correlations, and system-level validation should test whether predicted boron inventory, reactivity response and IVR/core-catcher margins remain consistent across independent reactor-relevant datasets.

### 5.3. Conclusions

This focused narrative review has examined recent progress in B_4_C–stainless-steel eutectic interaction for SFR severe-accident analysis, together with foundational work required to interpret the evidence. The following conclusions are drawn.

(1) At the local, unirradiated material-couple scale, B_4_C–SS counter-diffusion, boride-layer formation and interfacial liquefaction are supported by multiple experimental methods. Quantitative rates are not universal; however, heating rate, B_4_C powder/granule/pellet form, contact state, layer definition and liquid convection can shift both onset and fitted kinetics. Metallic-fuel FCCI is retained as a distinct contextual branch for metallic-fueled SFRs.
(2) BWR/LWR evidence is conditionally useful rather than uniformly transferable. Non-oxidizing local mechanisms and composition-matched property data can support SFR models after parameter matching; rod/bundle observations and simulants are most defensible as mechanism/model-validation evidence; and steam-oxidation results are not baseline in-vessel SFR inputs. Sodium, irradiation, bundle interactions and multicomponent fuel-bearing melts remain insufficiently validated.
(3) For safety analysis, the most consequential uncertainty is how B_4_C reaction extent controls mobile boron and melt composition, because these quantities jointly set recriticality margins and the thermophysical boundary conditions for relocation and IVR. Particle-method and integral-code studies show sensitivity to transport closures and compact material-interaction choices, but predictive use requires independently measured parameters, resolution convergence and propagated uncertainty.
(4) The priority path is therefore to quantify B_4_C–SS kinetics and properties under sodium-relevant, transient and increasingly prototypic conditions; close the multicomponent thermodynamic/transport database; validate mechanistic models on withheld experiments; and carry the resulting parameter distributions into SFR recriticality and IVR calculations.

## Figures and Tables

**Table 1:** Kinetic determinations for B_4_C–stainless steel interaction and the variables governing comparability.

Regime/Method	Materials, Form, Atmosphere	Heating Condition/T	Measured Quantity and Kinetic Form	Reported Parameters/Modifiers	Comparability/Uncertainty	Ref.
Solid–solid compatibility	B_4_C/Type 316 SS (1.4919); planar couple	Isothermal; 1073–1473 K (B_4_C/SS)	Reaction-zone growth; x^2^ = kt below extensive liquefaction	x^2^/t = 8.76 × 10^6^ exp(−378 kJ mol^−1^/RT) cm^2^ s^−1^ (1073–1473 K); rapid B_4_C/SS liquefaction above ~1523 K	Planar diffusion-couple result; geometry/form dependence not captured	[10]
Solid–solid annealing	B_4_C pellet/powder/Type 304 SS; Ar	Isothermal; 1073–1623 K	SS thinning and reaction-layer growth; parabolic k	Pellet: Ea = 250 kJ mol^−1^ (1073–1473 K) and 549 kJ mol^−1^ (1498–1623 K); powder: 283 and 453 kJ mol^−1^; ~2 orders increase at 1473–1498 K	Two regimes; measured SS side differs from immersion B_4_C-side definition	[19]
Granular control-rod geometry	Granular B_4_C/Type 304L SS; Ar	1453–1523 K; 4–240 min	Maximum reaction-layer thickness; k = x^2^/t	k = (5.88 ± 0.80) × 10^10^ exp[−(6.23 ± 2.79) × 10^5^/(RT)] m^2^ s^−1^	~1 order lower than pellet/powder data; large Ea uncertainty reflects contact scatter	[39]
DTA/Kissinger	B_4_C and Type 316L SS pellets; Ar 100 mL min^−1^	2.5, 5, 10 K min^−1^; onset 1483–1534 K	First endothermic onset; first-order Kissinger analysis	Ea = 511 ± 119 kJ mol^−1^; ln(A/s^−1^) = 34.6 ± 9.7	Kinetic event is DTA onset, not a layer-growth thickness	[23]
Rapid heating	B_4_C–Type 316L SS	80–110 K min^−1^; onset 1476–1503 K	Onset temperature and diffusion-layer thickness	Layer thickness 1.64 → 1.13 mm as heating rate increased	Heating-rate dependence is an explicit modifier	[32]
Solid–liquid immersion	Sintered B_4_C pellet in molten Type 316 SS; Ar	Isothermal 1763, 1853 and 1943 K; ~30 s immersion	B_4_C pellet-thickness reduction; kIM = xIM^2^/tIM	kIM = 3.6 × 10^−9^–5.3 × 10^−8^ m^2^ s^−1^; ≥2 orders below extrapolated solid–solid constants	B_4_C-side definition and convective liquid regime are not commensurate with SS-side solid-state k	[24]
Molten-pool dissolution	B_4_C/molten Type 316L SS; Ar or Ar–H_2_O	1723 K	Dissolution rate and interfacial phases	H_2_O effect not detected; C precipitation slows dissolution	Single temperature; interfacial carbon changes effective rate	[36]
Joule-heating visualization	B_4_C–Type 304 SS tubes; powder and pellet cases; Ar	Local electrical heating; 1450–1550 K	Optical reaction-layer growth	Powder reacts faster than pellet	Electrical heating and contact morphology differ from furnace/radiative conditions	[25,37]

**Table 3:** Recommended thermophysical-property data for B_4_C-bearing stainless steel used in relocation models.

Property	Composition/Phase	Temperature Range	Recommended Data/Correlation	Reported Uncertainty	Model Relevance	Ref.
Solid density, Cp, thermal conductivity	SS–5 mass% B_4_C; solid	293–1273 K	ρ = 7538 − 0.31955T kg m^−3^ (T in K); Cp and thermal conductivity from the fitted correlations in the source	Instrument error within ±5% (Archimedes density ±1%)	Pre-melting thermal response	[26]
Liquidus temperature	Type 316L SS/SS–5 mass% B_4_C	near transition	1719 ± 2 K/1608 ± 15 K	±2 K/±15 K	Phase-change criterion	[27]
Solidus/liquidus vs. composition	Type 316 SS–x mass% B_4_C, 0 ≤ x ≤ 10	Solid–liquid transition	Solidus 1666 K (x = 0) → 1307 K (x = 10), monotonic; liquidus minimum near x ≈ 2.5	As reported in source	Phase-change criterion for evolving melt composition	[14]
Liquid density ρ	SS–5 mass% B_4_C; liquid	1351–1907 K	ρ [kg m^−3^] = −0.716(T/K − 1608) + 6759	±1.5% expanded (95% confidence)	Buoyancy, stratification, momentum	[27]
Normal spectral emissivity ε	SS–5 mass% B_4_C; liquid	1334–1892 K	ε = 0.22 ± 0.01 (at 940 nm)	±0.01 (2σ as reported)	Radiative heating/pyrometry	[27]
Heat capacity Cp	SS–5 mass% B_4_C; liquid	1446–1824 K	Cp = 775 ± 48 J kg^−1^ K^−1^	±48 J kg^−1^ K^−1^ (2σ)	Energy/enthalpy balance	[27]
Thermal conductivity λ	SS–5 mass% B_4_C; liquid	1554–1838 K	λ = 33.8 ± 2.5 W m^−1^ K^−1^	±2.5 W m^−1^ K^−1^ (2σ)	Pool heat transfer	[27]
ε, Cp and λ vs. composition	Type 316 SS–x mass% B_4_C, 0 ≤ x ≤ 10; liquid	Wide liquid range (see source)	Composition-dependent data in source; trends change across the ≈3 mass% eutectic composition	See source	Energy balance and radiative heating for evolving composition	[59]
Surface tension	Type 316L SS–B_4_C; liquid	Wide liquid range; 0–10 mass% B_4_C in composition series	Use composition-specific levitation data; specify S/O impurity level	~±5% in 5 mass% study; impurity contribution additional	Wetting, breakup, capillary flow	[14,27]
Viscosity	Type 316L SS + 0–7.0 mass% B_4_C; liquid	1613–1793 K (composition-dependent); correlation fitted for 2.5–7.0 mass% at 1713–1793 K	Oscillating-crucible data and composition-dependent correlation in source; viscosity increases with B_4_C content	<±8% measurement error	Relocation velocity, stratification, particle methods	[58]

**Table 4:** Experimental relevance matrix for eutectic-melting and relocation studies used in the SFR-centered synthesis.

Approach	Reactor Context/Scale	Materials/Steel Grade	Atmosphere/Coolant; Heating	Primary Observables	What it Can Validate	Primary Limitations	Refs.
SCARABEE-N integral	SFR; in-pile subassembly	Fuel, cladding, structures	Sodium/SFR environment; blockage transient	Degradation/propagation/relocation	Direct SFR integral phenomenology	Older program; limited isolation of B_4_C-specific kinetics	[60]
BWR/LWR integral bundles	BWR/LWR; bundle	B_4_C absorber with fuel/cladding structures	Steam/reactor-specific; transient heating	~1523 K absorber failure, relocation, bundle collapse	Mechanistic sequence/scale effects	Steam oxidation, geometry and materials differ from SFR	[61,62,63,64,65,66,67,68]
Furnace/DTA couples	Separate effect; coupon/pellet	B_4_C with Type 304/316(L) SS	Ar/inert; isothermal or 2.5–110 K min^−1^	Layer growth, onset, kinetic parameters	Intrinsic diffusion/onset under controlled contact	Small scale; no sodium/irradiation; contact-form dependence	[10,19,23,32,39]
Immersion/molten-pool dissolution	Separate effect; pellet in melt	B_4_C pellet/molten SS	Controlled atmosphere; isothermal 1723–1943 K	B_4_C thinning/dissolution, interfacial phases	Solid–liquid transport and carbon effects	Reaction-side definition differs from solid-state data	[24,36]
Joule-heating visualization	Rod/couple scale	B_4_C powder/pellet with SS tube	Controlled gas; localized electrical heating	Optical layer growth, morphology effect	Short-time kinetics and local reaction-zone evolution	Heating-field bias; limited geometry; no sodium/irradiation	[25,37]
Radiative-heating visualization	SFR-oriented single rod	B_4_C powder/pellet/Type 304 SS	Ar; external radiation, 1423–1645 K	Failure mode, candling, melt phase, B transport	Externally heated relocation and rod-scale model comparison	Single rod; no sodium/irradiation; limited inter-facility replication	[13,53,54,70]
Simulant contact melting	Separate effect; coupon/pool	Pb–Sn, Bi–Pb	Controlled furnace/gas; 463–578 K	Parametric rates vs. T, pressure, geometry	Transport/contact algorithms and sensitivity trends	Cannot validate boride chemistry, phase formation or boron redistribution	[71,72,73]

**Table 5:** Transferability hierarchy for BWR/LWR- and laboratory-derived evidence used in SFR analysis.

Category	Examples	Permitted Use in SFR Analysis	Required Matching/Qualification
Directly transferable at material-couple level	Non-oxidizing phase identity; diffusion-layer mechanism	Mechanism definition and local thermodynamic constraints	Steel composition; B_4_C chemistry; temperature; phase state
Conditionally transferable after parameter matching	Kinetic constants; dissolution rates; thermophysical properties	Quantitative input within validated range	B_4_C form/contact, grade, heating rate, reaction-side definition, convection, composition, impurities, irradiation
Qualitative/model-validation only	BWR rod/bundle relocation; SFR single-rod radiative tests; simulants	Mode classification, code verification/validation targets, sensitivity trends	Geometry, scale, heating mode, coolant, wetting/capillarity, contact pressure and dimensionless transport regime
Not transferable to baseline in-vessel SFR	Steam oxidation/H_2_ generation/oxide-layer boron retention	Only oxidant-ingress, ex-vessel or post-relocation scenarios	A physically credible oxidizing environment must be demonstrated

**Table 6:** Structured comparison of particle-method models for eutectic melting and relocation.

Method/Study	Governing Physics & Phase Change	Mass Diffusion/Convection	Surface Tension & Viscosity	Thermodynamic Input	Fitted Parameters/Resolution Issue	Verification/Validation Status	Scale/Intended Use
MPS [17]	Lagrangian flow + energy; binary phase diagram	Fickian diffusion; flow resolved	Standard MPS closures	Pb–Sn phase diagram	Literature diffusion coefficients as input; resolution sensitivity not reported	Compared with Pb–Sn experiment; no blind validation	Simulant contact melting
MPS [18]	Energy + latent heat; phase-diagram melting	Effective diffusion	MPS closures	U–Fe phase diagram	Effective transport inputs	Verified vs. 1D analytical melting; compared with TREAT evidence	Mechanistic U–Fe penetration
Improved MPS [30]	Free-surface flow + eutectic criterion	B diffusion; relocation flow	Revised surface tension and viscosity	Fe–B criterion/experiment-informed	Effective diffusion; inter-particle spacing	Compared with radiative B_4_C–SS tests; coefficients not fully independent	Rod-scale B_4_C–SS relocation
SPH [31]	Incompressible SPH + heat transfer	Fickian diffusion + resolved convection	Explicit viscosity and surface-tension models	B_4_C–SS phase diagram; calibration to immersion data	D fitted vs. T; particle-size sensitivity	Calibration/reproduction of [24], not independent validation	Solid B_4_C in liquid SS; convection effect

**Table 7:** Compact material-interaction representations in severe-accident codes discussed in this review.

Code	Reactor Context	Material-Interaction Representation	Key State Variables/Inputs	Tunable or Calibrated Quantities	Evidence/Validation Basis	Documented Sensitivity
MELCOR V2.2	LWR/BWR	Eutectics model and interactive-materials option within core-degradation framework	Component temperatures, oxidation/material inventories, interaction criteria	Model option and interaction parameters	BWR-3 Mark-I Fukushima-representative benchmark [81,82]	Changes accident progression, lower-head debris state and H_2_ generation
SAS4A	Metallic-fuel SFR	Metallic fuel/cladding relocation with FCCI/eutectic interaction treatment	Fuel/cladding composition, temperature, relocation state	Interaction/relocation correlations derived from metallic-fuel evidence	Integral validation against eight TREAT M-Series overpower tests [5]	Affects cladding failure and relocation timing/composition
ASTEC	LWR/BWR	Integral core degradation/corium/material-interaction models	Core material inventories, oxidation, melt composition and relocation	Material-interaction/oxidation model parameters	Transient simulations of Fukushima Daiichi core-melt composition [83]	Predicted core-melt composition and degradation sequence; eutectic-model sensitivity not isolated here
EUCLID/V2 + HEFEST-FR	SFR/core catcher	2D heat conduction with melting/retention of catcher materials; receives relocated-melt conditions	Melt arrival temperature/composition; catcher properties	Thermophysical/catcher parameters and boundary conditions	SFR core-catcher analyses [16]	Affects retention/cooling margin; not a detailed B_4_C reaction model

## Data Availability

No new data were created or analyzed in this study. Data sharing is not applicable to this article.

## Abbreviations

The following abbreviations are used in this manuscript:

**Abbrev.**	**Definition**
ACRR	Annular Core Research Reactor
AES	Auger electron spectroscopy
ASTEC	Accident Source Term Evaluation Code
B_4_C	Boron carbide
BSAF	Benchmark Study of the Accident at the Fukushima Daiichi Nuclear Power Station
BWR	Boiling water reactor
CALPHAD	CALculation of PHAse Diagrams
CDA	Core disruptive accident
CEFR	China Experimental Fast Reactor
CFD	Computational fluid dynamics
CORA	CORA severe-fuel-damage/bundle-degradation test programme
CRIEPI	Central Research Institute of Electric Power Industry
DTA	Differential thermal analysis
EBR-II	Experimental Breeder Reactor-II
EBSD	Electron backscatter diffraction
EDS	Energy-dispersive spectroscopy
ELSA-1	Experiment on Late In-vessel Severe Accident Phenomena-1
EPMA	Electron probe microanalysis
EUCLID/V2	EUCLID integrated computer code, version 2
FCCI	Fuel–cladding chemical interaction
FDNPS	Fukushima Daiichi Nuclear Power Station
HEFEST-FR	HEFEST-FR fast-reactor/core-catcher module of EUCLID/V2
HT-9M	Modified HT9 ferritic/martensitic cladding steel
IVR	In-vessel retention
JAEA	Japan Atomic Energy Agency
JSFR	Japan Sodium-cooled Fast Reactor
LWR	Light water reactor
MCR	Multivariate curve resolution
MELCOR	MELCOR severe-accident analysis code
MPS	Moving Particle Semi-implicit method
OECD/NEA	Organisation for Economic Co-operation and Development/Nuclear Energy Agency
PGSFR	Prototype Gen-IV Sodium-Cooled Fast Reactor
PWR	Pressurized water reactor
PXRD	Powder X-ray diffraction
SEM	Scanning electron microscopy
SEM-EDS	Scanning electron microscopy with energy-dispersive spectroscopy
SFR	Sodium-cooled fast reactor
SPH	Smoothed particle hydrodynamics
SS	Stainless steel
TAF-ID	Thermodynamics of Advanced Fuels-International Database
TCFE12	Thermo-Calc steel/Fe-alloys database, version 12
TG-DTA	Thermogravimetry–differential thermal analysis
TREAT	Transient Reactor Test Facility
ULOF	Unprotected loss-of-flow
UO_2_	Uranium dioxide
UTOP	Unprotected transient overpower
WDS	Wavelength-dispersive X-ray spectroscopy
XMA	X-ray microanalysis
XPS	X-ray photoelectron spectroscopy
XR2-1	BWR metallic-melt relocation experiment XR2-1
XRD	X-ray diffraction
